# On Exploiting Millimeter-Wave Spectrum Trading in Countrywide Mobile Network Operators for High Spectral and Energy Efficiencies in 5G/6G Era

**DOI:** 10.3390/s20123495

**Published:** 2020-06-20

**Authors:** Rony Kumer Saha

**Affiliations:** Radio and Spectrum Laboratory, KDDI Research Inc., 2-1-15 Ohara, Fujimino-shi, Saitama 356-8502, Japan; ro-saha@kddi-research.jp

**Keywords:** 28 GHz, 6G, countrywide, exclusive-use, millimeter-wave, method, mobile network, small cells, spectrum access, spectrum trading

## Abstract

In this paper, we propose a dynamic exclusive-use spectrum access (DESA) method to improve the overall licensed millimeter-wave (mmWave) spectrum utilization of all mobile network operators (MNOs) in a country. By exploiting secondary spectrum trading, the proposed DESA method shares partly and exclusively the licensed mmWave spectrum of one MNO to another in a dynamic and on-demand basis for a certain agreement term. We formulate the proposed DESA method for an arbitrary number of MNOs in a country. We then present an iterative algorithm to find the optimal amount of shared spectrum for each MNO, which is updated at each agreement term. We derive average capacity, spectral efficiency, energy efficiency, and cost efficiency performance metrics for all MNOs countrywide and present extensive numerical and simulation results and analyses for an example scenario of a country with four MNOs each assigned statically with an equal amount of 28-GHz mmWave spectrum. By applying DESA, we show that MNOs with a lack of minimum licensed spectra to serve their data traffic can lease at the cost of payment of the required additional spectra from other MNOs having unused or under-utilized licensed spectra. Moreover, it is shown that the overall countrywide average capacity, spectral efficiency, energy efficiency, and cost efficiency can be improved, respectively, by 25%, 25%, 17.5%, and 20%. Furthermore, we show that, by applying DESA to all MNOs countrywide, the expected spectral efficiency and energy efficiency requirements for sixth-generation (6G) mobile systems can be achieved by reusing the same mmWave spectrum to 20% fewer buildings of small cells. Finally, using the statistics of subscribers of all MNOs, we present a case study for fifth-generation (5G) networks to demonstrate the application of the proposed DESA method to an arbitrary country of four MNOs.

## 1. Introduction

### 1.1. Background

The number of users of mobile communications continues to rise every decade. Although the subscriber-base of a mobile network operator (MNO) has been increased manifold in the last decade, the radio spectrum allocated to an MNO has not been increased in proportion [1]. Moreover, the majority of the available spectrum is allocated to MNOs of a country statically on a long-term basis to cover large geographical areas. Such a static allocation with a dedicated licensed spectrum to an MNO is no longer sufficient to serve enormous data traffic demands of existing users. Moreover, the static allocation of spectrum causes large portions of licensed spectrum per MNO to be under-utilized at different times and geographic locations [2,3,4], with an average percentage of licensed spectrum usage ranging from 15% to 85% [5,6].

Hence, due to the scarcity and under-utilization of the available licensed spectrum, along with the high cost of licensing, efficient spectrum utilization is crucial to serve the ever-increasing demand for network capacity, as well as to provide high quality-of-service (QoS) at a low cost per bit transmission [7], mostly in indoor coverage. In this regard, to improve the spectrum utilization, dynamic spectrum access models such as shared-use model and exclusive-use model have been proposed in the literature for the dynamic and on-demand basis access to the licensed spectra.

Numerous research works have addressed the spectrum sharing issue. For example, the authors of [8] studied the main concepts of dynamic spectrum sharing, different sharing scenarios, along with major challenges associated with the sharing of licensed bands. In [9], the authors studied spectrum sharing approaches, as well as user association mechanisms, in mmWave systems. Likewise, the authors of [10] discussed some key spectrum management challenges (related to the dynamic spectrum access at the mmWave spectrum) that mus be overcome to promote dynamic spectrum sharing at the mmWave spectrum bands. Further, the authors of [11] characterized the rate complementary cumulative distribution function for a spectrum-shared mmWave network where multiple operators share mmWave bandwidths with each other while using dynamic inter-operator base station coordination to suppress cross-operator interference. Furthermore, in [12], the authors introduced a new hybrid spectrum access scheme for mmWave networks where data packets are scheduled through two mmWave carriers with different characteristics.

However, due to going against the interest of primary users’ (PUs’) licensed spectra by secondary users (SUs) in the shared-use model [2], the exclusive-use model is considered as one of the efficient models to improve licensed spectrum utilization. In exclusive-use access models, the under-utilized licensed spectrum allocated statically to primary service providers (PSPs) can be leased to secondary service providers (SSPs) exclusively for a certain duration of time. Such exclusive access of spectrum in cognitive networks is termed as secondary spectrum trading [13], which could happen either by PSPs and SSPs directly between each other or by a spectrum broker.

Spectrum trading is an important feature of spectrum sharing, which considers both commercial and technical aspects [14,15]. Spectrum trading as a secondary mechanism allows primary players with dedicated licensed spectra to trade in full or part their licensed spectra with other secondary players by transferring them exclusively the licensed spectrum usage rights of primary players for a certain duration of time [16]. Either macro factors such as demand and technology or micro factors such as the economically inefficient assignment of the primary spectrum and changes in strategy and service of a company may cause spectrum trading [17]. Regardless of the primary spectrum allocation mechanisms, the secondary spectrum trading always plays an active role for efficient spectrum utilization [7]. A major advantage of the dynamic exclusive-use model is that multiple buyers and sellers can be benefitted from the secondary spectrum trading: sellers can make additional revenue by selling their unused licensed spectrum, whereas buyers can have required additional guaranteed-spectrum access to fulfill their user demands for a certain duration of time [2].

### 1.2. Related Work

Numerous research studies have already addressed spectrum trading from various aspects. For example, the authors of [18] proposed a matching based double auction mechanism for spectrum trading with differential privacy to protect the privacy of buyers/sellers from the untrustworthy auctioneer and other buyers/sellers and potential parties. In [19], the authors proposed a privacy-preserving secure spectrum trading and sharing scheme between the aerial and terrestrial communication systems based on blockchain technology. Further, the authors of [20] formulated spectrum trading problems by means of contract theory for a macro base station (BS) of a terrestrial operator and unmanned aerial vehicle (UAV) operators given that the manager of the macro BS has to design an optimal contract to maximize its revenue.

The authors of [21] proposed a two-tier spectrum trading strategy that includes two trading processes such that, in Process 1, the spectrum trading is modeled as a monopoly market, whereas, in Process 2, the spectrum trading is modeled as a multi-seller, multi-buyer market. In [22], the authors introduced a bandwidth-auction game for the spectrum trading problem of a cellular network consisting of multiple cellular user equipments (UEs) as the primary users and a cognitive device-to-device (D2D) pair as the secondary user. Further, in [23], the authors studied the spectrum trading system from the service-oriented perspective considering all three aspects on secondary spectrum licensees, spectrum market, and primary spectrum licensees to promote the implementation of trading-based licensed spectrum sharing.

In addition, several research works have addressed spectrum trading using tools such as game theory to analyze and model interactive decision-making processes [24] for non-auction and monetary-based approaches. For example, in [25], the authors modeled the competition for the spectrum access among multiple SUs from a single PU using non-cooperative game theory. In [26], authors considered two secondary operators as spectrum sellers who lease spectrum from the spectrum owners and sell those spectra to multiple SUs. Besides several works have addressed spectrum trading for multiple sellers and multiple buyers’ scenarios. For example, the authors of [27] considered multiple sellers and multiple buyers in a spectrum market where sellers compete with one another to set the price of the spectrum, and buyers select the spectrum based on either the quality or the price of the sellers. The authors of [28] also proposed a scheme using the evolutionary game theory for selling the spectra of multiple PUs to multiple SUs who update their strategies from time to time to maximize transmission rate and price payoffs.

Moreover, in [29], the authors considered a differential game to model the competition among multiple SSPs of a scheme where SSPs can lease the spectrum directly from the spectrum brokers usually for a short term to sell those spectra to the SUs. Further, in [30], with an agent, the authors considered spectrum trading among multiple PSPs and SUs and provided a solution using market equilibrium given that the agent submits the collected spectrum demands from SUs to the PSPs. Furthermore, a recall-based spectrum trading was considered by the authors of [31] such that an SU can receive compensation from the PSPs for the amount of spectrum it recalled determined using the Stackelberg game. However, exploiting the spectrum trading to improve the overall licensed millimeter-wave (mmWave) spectrum utilization of all MNOs of a country is not *obvious* in existing literature, which we address in this paper.

### 1.3. Contribution

To address that, we propose a dynamic exclusive-use spectrum access (DESA) method that allows sharing partly and exclusively the 28 GHz licensed mmWave spectrum of one MNO to another of a country in a dynamic and on-demand basis for a certain time of their mutual agreement to improve countrywide spectrum utilization. We formulate the problem for the proposed DESA method for an arbitrary number of MNOs in a country each assigned statically with the 28 GHz mmWave spectrum. An iterative algorithm is then presented to find the optimal amount of shared spectrum for each MNO at each agreement term. With applying DESA, we derive performance metrics, including average capacity, spectral efficiency (SE), energy efficiency (EE), and cost efficiency (CE), for all MNOs countrywide. Extensive numerical and simulation results and analyses for an example scenario of a country with four MNOs each assigned statically with an equal amount of 28 GHz mmWave spectrum is presented to show the overall countrywide average capacity, SE, EE, and CE improvement. Moreover, we show that, with applying DESA, the expected SE and EE requirements for sixth-generation (6G) mobile systems can be achieved by reusing the same mmWave spectrum to fewer buildings of small cells. Finally, we present a case study for fifth-generation (5G) mobile networks to demonstrate the application of the proposed DESA method to an arbitrary country of four MNOs.

### 1.4. Organization

The paper is organized as follows. We present the system architecture, the proposed DESA method, and an iterative algorithm in Section 2. In Section 3, the average capacity, SE, EE, and CE performance metrics are derived. Numerical and simulation results and analyses are presented in Section 4. In Section 5, we discuss the performance comparison for the proposed DESA method relating to satisfying the SE and EE requirements for 6G mobile systems, as well as a case study for 5G networks demonstrating the use of DESA to an arbitrary country of four MNOs. We conclude the paper in Section 6.

## 2. System Architecture and Proposed Method

### 2.1. System Architecture

**Figure 1:**
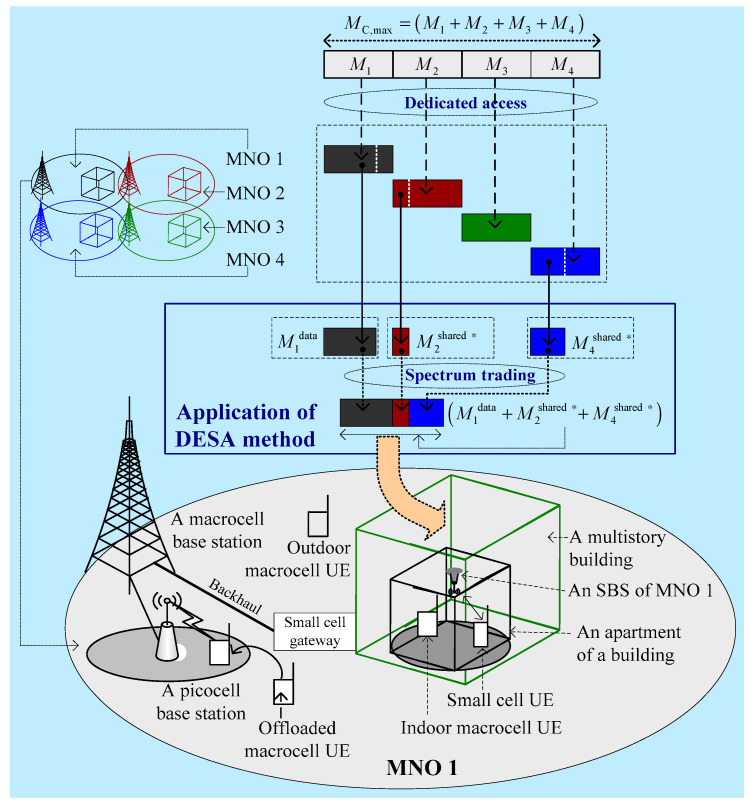
System architecture for four MNOs of a country applying the proposed DESA method. $M_{C,\max}$ denotes the total amount of 28 GHz licensed mmWave spectrum countrywide. $M_{1}$, $M_{2}$, $M_{3}$, and $M_{4}$ denote, respectively, the mmWave spectrum of MNO 1, MNO 2, MNO 3, and MNO 4. $M_{1}^{\mathrm{data}}$ denotes data traffic spectrum of MNO 1, whereas $M_{4}^{\mathrm{shared}}{\;}^{*}$ and $M_{2}^{\mathrm{shared}}{\;}^{*}$ denote leased spectrum from MNO 2 and MNO 4, respectively, such that they constitute the total mmWave spectrum of MNO 1 with applying DESA due to spectrum trading.

Consider that there are *O* MNOs operating in a country, each consisting of three types of BSs: macrocell BSs (MBSs), picocell BSs (PBSs), and small cell BSs (SBSs). Figure 1 shows the system architecture for *O* = 4. For simplicity, we show only one macrocell per MNO in Figure 1. Moreover, we assume that all MNOs have similar system architectural features such that the detailed architecture, including the application of the proposed DESA method, only for MNO 1 is shown in Figure 1. Note that, in regard to applying DESA, we assume that MNO 1 lacks a sufficient amount of spectrum to serve its data traffic such that it leases the corresponding amount of spectrum from MNO 2 and MNO 4 (Figure 1). We discuss in detail the proposed DESA method in the following section.

As shown in Figure 1, within the coverage of a macrocell, picocells and small cells are deployed. We assume that small cells are located only within three-dimensional (3D) multistory buildings. Both the macrocell and picocells operate at the same 2 GHz microwave spectrum band, whereas all small cells per building operate only at the 28 GHz mmWave spectrum band due to the favorable propagation characteristics of high-frequency mmWave signals in indoor environments. A certain percentage of macrocell UEs is considered indoor within buildings. Further, several macrocell UEs are considered offloaded to picocells. All macrocell UEs are served by either the macrocell or any picocell. However, small cells serve only their own indoor small cell UEs to provide good indoor coverage. 

### 2.2. Proposed Method

#### 2.2.1. Principle

We propose a dynamic exclusive-use spectrum access (DESA) method to share partly and exclusively the licensed mmWave spectrum of one MNO to another of a country on-demand basis stated as follows: *Each MNO with a licensed mmWave spectrum can share exclusively a part of its spectrum mutually with other MNOs of a country in a dynamic and on-demand basis for a certain time of their mutual agreement to improve countrywide spectrum utilization, subject to updating the shared spectrum of each MNO at each agreement term.*

The proposed DESA method stated above considers exploiting the secondary spectrum trading such that, unlike the individual authorization (i.e., licensed or static access) method, the licensed spectrum of an MNO can be used by other licensed MNOs of a country, usually for a short duration, with mutual understanding between MNOs. Further, the shared or leased spectrum by an MNO can be given back to the original licensee after the agreement term if the shared spectrum is found unused or under-utilized. In addition, usually, a portion of the licensed spectrum of an MNO is shared with other MNOs to dynamically meet user demands of both MNOs by periodically revising the shared spectra of each MNO at each agreement term.

Similarly, unlike the general authorization (i.e., license-exempt) method, use of the shared spectrum is secured by enforcing protection such that the secondary MNO (s-MNO), i.e., the MNO with the shared spectrum, cannot cause interference to the primary MNO (p-MNO), i.e., the MNO who shares its spectrum at an agreement term. Further, the s-MNO pays for the leased spectrum to the p-MNO only for the portion of the spectrum that it uses under the secondary spectrum trading. Furthermore, since each MNO is also granted a license for the mmWave spectrum using the static spectrum access method, coordination is necessary between the p-MNOs and s-MNOs to share the spectrum of the p-MNO with the s-MNO to fill the spectrum scarcity of the s-MNO, due to a surge in the user demand of the s-MNO at any agreement term, for example.

Based on the above discussion, the proposed DESA method can be viewed as the light licensing spectrum access method, which combines advantages of both opposing licensing methods, namely individual authorization and general authorization, to allow a more flexible, simplified, and interference-protection secured way for sharing the mmWave spectrum of one MNO to another. It is to be noted that the light licensing spectrum access method is mainly proposed for spectrum bands with low interference risk and high capacity, namely mmWave spectrum bands. A list of notations is given in Table A1 in Appendix A.

#### 2.2.2. Problem Formulation

Let *O* denote the maximum number of MNOs of a country such that o∈O:O={1,2,…,O}. Let $M_{C,\max}$ denote the total amount of mmWave spectrum in terms of the number of resource blocks (RBs) allocated to a country where an RB is equal to 180 kHz. Assume that each MNO is licensed initially (i.e., *t*_agg_ = 0) a portion of $M_{C,\max}$ denoted as $M_{o,t_{\mathrm{agg}}=0}$ where $t_{\mathrm{agg}}$ denotes an agreement term to update the amount of spectrum of any MNO by trading with other MNOs such that at any $t_{\mathrm{agg}}$, $\sum_{o=1}^{O}M_{o,t_{\mathrm{agg}}}\leq M_{C,\max}$ holds. To address fairness in the static spectrum allocation, assume that each MNO of the country is allocated initially at *t*_agg_ = 0 an equal amount of mmWave spectrum of *M* RBs. Note that $M_{C,\max}$ does not change with $t_{\mathrm{agg}}$ and is typically fixed for a long time. Assume that $M_{o,t_{\mathrm{agg}}}^{\mathrm{res}}$ denotes the reserved spectrum in RBs of an MNO *o* to serve its control signaling, emergency purposes, and other system-specific requirements at any agreement term $t_{\mathrm{agg}}$.

Note that the available data traffic spectrum in RBs for an MNO *o* at any $t_{\mathrm{agg}}$ is the data traffic spectrum updated in the previous agreement term $t_{\mathrm{agg}}-1$ (i.e., $M_{o,t_{\mathrm{agg}}-1}^{\mathrm{data}}$), whereas the required data traffic spectrum in RBs for an MNO *o* at $t_{\mathrm{agg}}$ is given by $M_{o,t_{\mathrm{agg}}}^{\mathrm{data}}$. Assume that $N_{o,t_{\mathrm{agg}}}$ denotes the total number of subscribers for an MNO *o* at $t_{\mathrm{agg}}$ such that $\sum_{o}^{O}N_{o,t_{\mathrm{agg}}}\leq N_{C,\;\max,t_{\mathrm{agg}}}$ where $N_{C,\;\max,t_{\mathrm{agg}}}$ denotes the maximum number of subscribers of all MNOs of a country at $t_{\mathrm{agg}}$. Let $O_{b,t_{\mathrm{agg}}}$ and $O_{s,t_{\mathrm{agg}}}$ denote, respectively, the number of buyer MNOs and the number of seller MNOs at $t_{\mathrm{agg}}$. Since secondary spectrum trading is not free of cost, leasing more spectrum causes to increase the cost of the leased spectrum from a buyer MNO’s perspective, while to degrade the quality-of-service (QoS) from a seller MNO’s perspective. To address this problem, the seller MNO may want to lease its licensed spectrum as minimum as possible to the buyer MNO while ensuring to serve its user demands adequately to retain QoS. Likewise, the buyer MNO may want to take a lease of the licensed spectrum from the seller MNO as minimum as possible to reduce the cost of the leased spectrum. Since both the buyer MNO, as well as the seller MNO, favor to minimizing the amount of the leased spectrum, we consider a minimization problem for spectrum trading to increase the overall countrywide spectrum utilization. Then, the optimal amount of shared or leased spectrum $M_{o,t_{\mathrm{agg}}}^{\mathrm{shared}}$ in RBs for each MNO o∈O at any agreement term $t_{\mathrm{agg}}$ can be found by solving the following minimization problem.

(1) $$\begin{array}{ll} \underset{o\in O}{\min} & M_{o,t_{\mathrm{agg}}}^{\mathrm{shared}}\\ \mathrm{subject}\;\mathrm{to} & (a)\;\forall o\;M_{o,t_{\mathrm{agg}}=0}=M\\ & (b)\;\forall o\forall t_{\mathrm{agg}}\sum_{o}^{O}N_{o,t_{\mathrm{agg}}}\leq N_{C,\;\max,t_{\mathrm{agg}}}\\ & (c)\;\forall o\forall t_{\mathrm{agg}}\;\sum_{o=1}^{O}M_{o,t_{\mathrm{agg}}}\leq M_{C,\max}\\ & (d)\;\forall o\forall t_{\mathrm{agg}}M_{o,t_{\mathrm{agg}}}^{\mathrm{data}}=\;\left(M_{o,t_{\mathrm{agg}}}-M_{o,t_{\mathrm{agg}}}^{\mathrm{res}}\right)\\ & (e)\;\forall t_{\mathrm{agg}}O_{t_{\mathrm{agg}}}=O_{b,t_{\mathrm{agg}}}+O_{s,t_{\mathrm{agg}}}\; \end{array}$$

To solve the above problem, we consider an iterative algorithm as follows for finding the optimal amount of shared spectrum for each MNO iteratively at each agreement term. 

#### 2.2.3. Iterative Algorithm

Initially (i.e., at *t*_agg_ = 0), assume that each MNO is licensed exclusively an equal amount of mmWave spectrum of *M* in RBs satisfying Constraint 1(a) such that $\forall o\;M_{o,t_{\mathrm{agg}}=0}=M$. Then, the amount of effective spectrum in RBs to serve data traffic for each MNO at $t_{\mathrm{agg}}=0$ is given by,

(2) $$\forall o\;M_{o,t_{\mathrm{agg}}=0}^{\mathrm{data}}=\left(M_{o,t_{\mathrm{agg}}=0}-M_{o,t_{\mathrm{agg}}=0}^{\mathrm{res}}\right)$$

(3) $$\forall o\;M_{o,t_{\mathrm{agg}}=0}^{\mathrm{data}}=\left(M-M_{o,t_{\mathrm{agg}}=0}^{\mathrm{res}}\right)$$

Because, in general, the number of subscribers of one MNO differs from another, for simplicity, we assume that $N_{1,t_{\mathrm{agg}}}>N_{2,t_{\mathrm{agg}}}>\;\ldots >N_{O,t_{\mathrm{agg}}}$ at agreement term *t*_agg_ such that $\sum_{o}^{O}N_{o,t_{\mathrm{agg}}}\leq N_{C,\;\max,t_{\mathrm{agg}}}$ holds satisfying Constraint 1(b).

Applying Constraints 1(c) and 1(d), the total amount of effective spectrum in RBs for serving data traffic of all MNOs of a country at agreement term *t*_agg_ is then given by,

(4) $$M_{C,\mathrm{data},t_{\mathrm{agg}}}=\sum_{o=1}^{O}M_{o,t_{\mathrm{agg}}}^{\mathrm{data}}$$

(5) $$M_{C,\mathrm{data},t_{\mathrm{agg}}}=\sum_{o=1}^{O}\left(M_{o,t_{\mathrm{agg}}}-M_{o,t_{\mathrm{agg}}}^{\mathrm{res}}\right)$$

Recall that the amount of spectrum required to serve data traffic of an MNO *o* is proportional to its number of subscribers $N_{o,t_{\mathrm{agg}}}$ at *t*_agg_. Since the available data traffic spectrum in RBs for an MNO *o* at any $t_{\mathrm{agg}}$ is the data traffic spectrum updated in the previous agreement term $t_{\mathrm{agg}}-1$ (i.e., $M_{o,t_{\mathrm{agg}}-1}^{\mathrm{data}}$), the required data traffic spectrum in RBs for an MNO *o* at $t_{\mathrm{agg}}$ to serve data traffic of its subscribers $N_{o,t_{\mathrm{agg}}}$ can be found as follows.

(6) $$M_{o,t_{\mathrm{agg}}}^{\mathrm{data}}=\;\left(\frac{N_{o,t_{\mathrm{agg}}}\times M_{C,\;\mathrm{data},t_{\mathrm{agg}}-1}}{\sum_{o}^{O}N_{o,t_{\mathrm{agg}}}}\right):o\in O$$

(7) $$M_{o,t_{\mathrm{agg}}}^{\mathrm{data}}=\;\left(\frac{N_{o,t_{\mathrm{agg}}}\times \sum_{o=1}^{O}M_{o,t_{\mathrm{agg}}-1}^{\mathrm{data}}}{\sum_{o}^{O}N_{o,t_{\mathrm{agg}}}}\right):o\in O$$

(8) $$M_{o,t_{\mathrm{agg}}}^{\mathrm{data}}=\;\left(\frac{N_{o,t_{\mathrm{agg}}}\times \sum_{o=1}^{O}\left(M_{o,t_{\mathrm{agg}}-1}-M_{o,t_{\mathrm{agg}}-1}^{\mathrm{res}}\right)}{\sum_{o}^{O}N_{o,t_{\mathrm{agg}}}}\right):o\in O$$

Hence, the optimal amount of shared spectrum in RBs for an MNO *o*, being either a seller or a buyer of the shared spectrum, at an agreement term *t*_agg_ is given by,

(9) $$M_{o,t_{\mathrm{agg}}}^{\mathrm{shared}*}=\left(M_{o,t_{\mathrm{agg}}}^{\mathrm{data}}-M_{o,t_{\mathrm{agg}}-1}^{\mathrm{data}}\right):o\in O$$

(10) $$M_{o,t_{\mathrm{agg}}}^{\mathrm{shared}}{\;}^{*}=\;\left(\frac{N_{o,t_{\mathrm{agg}}}\times \sum_{o=1}^{O}\left(M_{o,t_{\mathrm{agg}}-1}-M_{o,t_{\mathrm{agg}}-1}^{\mathrm{res}}\right)}{\sum_{o}^{O}N_{o,t_{\mathrm{agg}}}}\right)-M_{o,t_{\mathrm{agg}}-1}^{\mathrm{data}}:o\in O$$

In the above expression, a positive value of $M_{o,t_{\mathrm{agg}}}^{\mathrm{shared}}{\;}^{*}$ implies that the corresponding MNO *o* lacks the required amount of spectrum given by $\left|M_{o,t_{\mathrm{agg}}}^{\mathrm{shared}}{\;}^{*}\right|$, and hence the MNO *o* is a buyer of the shared spectrum. On the contrary, $M_{o,t_{\mathrm{agg}}}^{\mathrm{shared}}{\;}^{*}$ is negative if the corresponding MNO *o* has an excess of spectrum given by $\left|M_{o,t_{\mathrm{agg}}}^{\mathrm{shared}}{\;}^{*}\right|$ even after serving its user demand such that the MNO *o* is a seller of the spectrum $\left|M_{o,t_{\mathrm{agg}}}^{\mathrm{shared}}{\;}^{*}\right|$, which can be shared with other MNOs who lack the required data traffic spectrum. Note that, to satisfy Constraints 1(b) and 1(c), based on the sign of $M_{o,t_{\mathrm{agg}}}^{\mathrm{shared}}{\;}^{*}$, an MNO *o* can either sell or buy the maximum spectrum given by $\left|M_{o,t_{\mathrm{agg}}}^{\mathrm{shared}}{\;}^{*}\right|$ from more than one MNOs such that $\sum_{o=1}^{O}M_{o,t_{\mathrm{agg}}}^{\mathrm{shared}}{\;}^{*}=0$.

**Figure 2:**
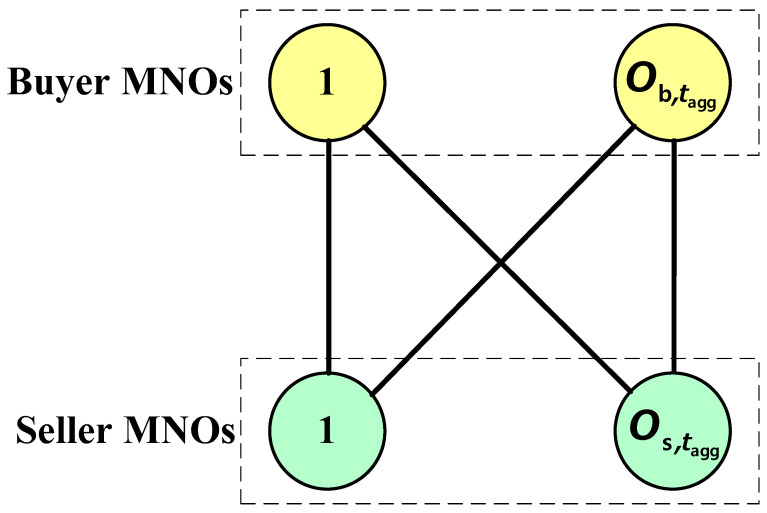
Spectrum trading among buyer and seller MNOs using DESA.

Besides, we assume that the spectrum trading using DESA among MNOs happens based on the mutual agreement, without the help of any spectrum broker. Based on the user demand and subject to satisfying Constraints 1(b) and 1(c), an MNO with a positive sign of $M_{o,t_{\mathrm{agg}}}^{\mathrm{shared}}{\;}^{*}$ (i.e., a buyer MNO) can trade only with MNOs with a negative sign of $M_{o,t_{\mathrm{agg}}}^{\mathrm{shared}}{\;}^{*}$ (i.e., seller MNOs) and vice versa at any *t*_agg_ to share the spectrum so long as a mutual agreement exists among MNOs. This can be presented by a complete bipartite graph as shown in Figure 2, which represents the relationship only between any buyer MNO and any seller MNO for leasing the spectrum at any *t*_agg_. However, there is no relationship either between buyer MNOs or between seller MNOs. Note that, in Figure 2, $O_{b,t_{\mathrm{agg}}}$ and $O_{s,t_{\mathrm{agg}}}$, respectively, denote the number of buyer MNOs and seller MNOs such that $O\;=\left(O_{b,t_{\mathrm{agg}}}+O_{s,t_{\mathrm{agg}}}\right)$ satisfying Constraint 1(e).

**Figure 3:**
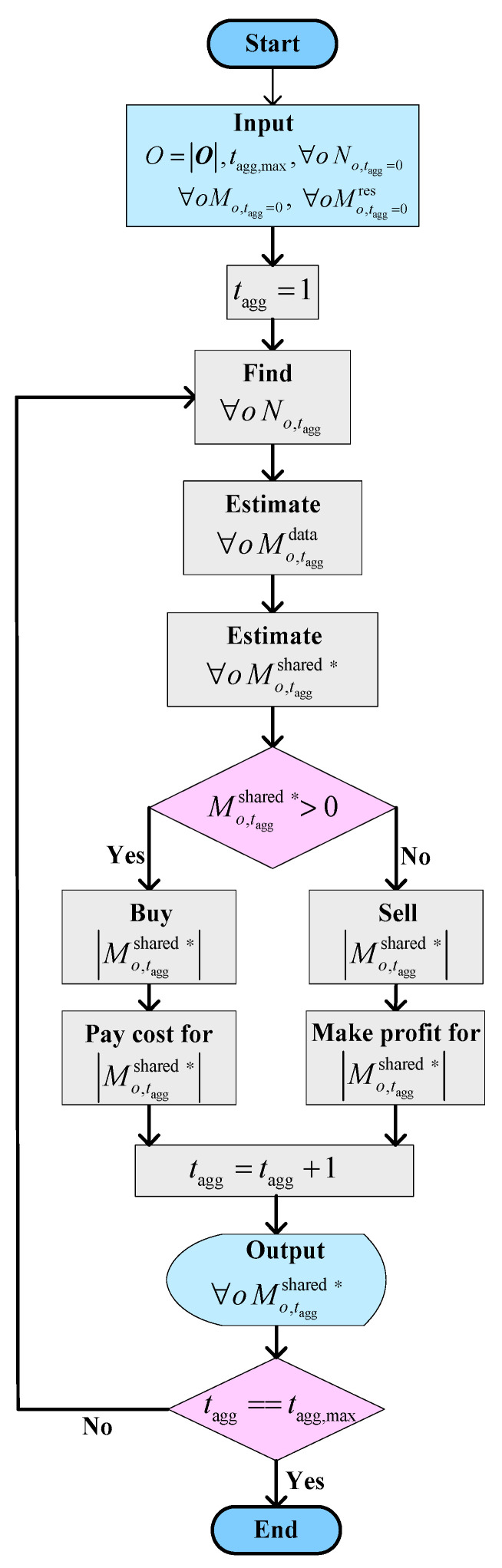
A flowchart of the proposed DESA method.

The same process described above is executed at each agreement term. If an MNO *o* with a positive sign of $M_{o,t_{\mathrm{agg}}}^{\mathrm{shared}}{\;}^{*}$ at the agreement term *t*_agg_ is found with a negative sign of $M_{o,t_{\mathrm{agg}}+1}^{\mathrm{shared}}{\;}^{*}$ in the next agreement term *t*_agg_+1, the MNO can take its shared spectrum $\left|M_{o,t_{\mathrm{agg}}}^{\mathrm{shared}}{\;}^{*}\right|$ at *t*_agg_ back from the corresponding MNO. Moreover, if an MNO *o* requires more spectrum (even after taking back its shared spectrum) to serve its user data traffic demand, the MNO *o* can buy additional spectra from other MNOs with a negative sign of $M_{o,t_{\mathrm{agg}}+1}^{\mathrm{shared}}{\;}^{*}$ at *t*_agg_ + 1. This process is applicable for all MNOs at each agreement term to update on-demand basis spectrum leasing among each other flexibly so that both the QoS as well as the profit gain from either the unused or the under-utilized spectrum can be achieved to improve the overall countrywide spectrum utilization. A flowchart of the algorithm for four MNOs is shown in Figure 3.

## 3. Mathematical Analysis

### 3.1. Preliminaries

For each MNO, assume that *L* denotes the number of buildings per macrocell coverage such that l∈{1,2,…,L}. Let *S*_F_ denote the maximum number of small cells per 3D building such that $s\in \left\{1,2,\ldots ,S_{F}\right\}$ where *S*_F_ is assumed the same for each of the *L* buildings. Let *S*_M_ denote the number of macrocells, and *S*_P_ denotes the number of picocells per macrocell of each MNO *o*. Assume that ***T*** denotes simulation run time with the maximum time of *Q* (in time step each lasting 1 ms) such that ***T***={1, 2, 3,…, *Q*}. In the following, performance metrics for system-level including all types of BSs and UEs, as well as only in-building SBSs and small cell UEs, are derived.

### 3.2. System-Level Performance

#### 3.2.1. Case 1: Single MNO

The downlink received signal-to-interference-plus-noise ratio for a UE at RB = *i* in the transmission time interval (TTI) = *t* for an MNO *o* at an agreement term *t*_agg_ can be expressed as

(11) $$\rho_{t,i,o}^{t_{\mathrm{agg}}}=\left(P_{t,i,o,t_{\mathrm{agg}}}/\left(N_{t,i,o,t_{\mathrm{agg}}}^{s}+I_{t,i,o,t_{\mathrm{agg}}}\right)\right)\;\times \;H_{t,i,o,t_{\mathrm{agg}}}$$

where $P_{t,i,o,t_{\mathrm{agg}}}$ is the transmission power, $N_{t,i,o,t_{\mathrm{agg}}}^{s}$ is the noise power, and $I_{t,i,o,t_{\mathrm{agg}}}$ is the total interference signal power. $H_{t,i,o,t_{\mathrm{agg}}}$ is the link loss for a link between a UE and a base station at RB = *i* in TTI = *t* for an MNO *o* at an agreement term *t*_agg_*,* which can be expressed in dB as

(12) $$H_{t,i,o,t_{\mathrm{agg}}}\left(\mathrm{dB}\right)=(G_{t}+G_{r})-(L_{F}+PL_{t,i,o,t_{\mathrm{agg}}})+(LS_{t,i,o,t_{\mathrm{agg}}}+SS_{t,i,o,t_{\mathrm{agg}}})$$

where $\left(G_{t}+G_{r}\right)$ and $L_{F}$ are, respectively, the total antenna gain and connector loss. $LS_{t,i,o,t_{\mathrm{agg}}}$, $SS_{t,i,o,t_{\mathrm{agg}}}$, and $PL_{t,i,o,t_{\mathrm{agg}}}$, respectively, denote large-scale shadowing effect, small-scale Rayleigh or Rician fading, and distance-dependent path loss between a base station and a UE at RB = *i* in TTI = *t* for an MNO *o* at an agreement term *t*_agg_.

Using Shannon’s capacity formula, a link throughput at RB = *i* in TTI = *t* for an MNO *o* at an agreement term *t*_agg_ in bps per Hz is given by [32,33],

(13) $$\sigma_{t,i,o}^{t_{\mathrm{agg}}}\left(\rho_{t,i,o}^{t_{\mathrm{agg}}}\right)=\left\{\begin{array}{ll} 0, & \;\rho_{t,i,o}^{t_{\mathrm{agg}}}<-10\;\mathrm{dB}\\ \beta \log_{2}\left(1+10^{\left(\rho_{t,i,o}^{t_{\mathrm{agg}}}\left(\mathrm{dB}\right)/10\right)}\right)\;, & -10\;\mathrm{dB}\leq \rho_{t,i,o}^{t_{\mathrm{agg}}}\leq 22\;\mathrm{dB}\\ 4.4, & \;\rho_{t,i,o}^{t_{\mathrm{agg}}}>22\;\mathrm{dB} \end{array}\right\}$$

where *β* denotes the implementation loss factor. 

Let *M*_MBS,*o*_ denote in RBs the operating spectrum of a macrocell for an MNO *o*. Then, the total capacity of all macrocell UEs serving at *M*_MBS,*o*_ spectrum for an MNO *o* at an agreement term *t*_agg_ can be expressed as

(14) $$\sigma_{\mathrm{MBS},o}^{t_{\mathrm{agg}}}=\sum_{t=1}^{Q}\sum_{i=1}^{M_{\mathrm{MBS},o}}\sigma_{t,i,o}^{t_{\mathrm{agg}}}\left(\rho_{t,i,o}^{t_{\mathrm{agg}}}\right)$$

where σ and ρ are responses over $M_{\mathrm{MBS},o}$ RBs of all macro UEs in *t*∈***T*** for an MNO *o* at term *t*_agg_.

The capacity served by an SBS of an MNO *o* at an agreement term *t*_agg_ is given by,

(15) $$\sigma_{s,o}^{t_{\mathrm{agg}}}=\sum_{t\in T}\sum_{i=1}^{M_{o,t_{\mathrm{agg}}}^{\mathrm{data}}}\sigma_{t,i,o}^{t_{\mathrm{agg}}}\left(\rho_{t,i,o}^{t_{\mathrm{agg}}}\right)$$

If all SBSs in each multistory building serves simultaneously in *t*∈***T***, then, the aggregate capacity served by all SBSs per building of an MNO *o* at an agreement term *t*_agg_ is given, respectively, by,

(16) $$\sigma_{S_{F},o}^{t_{\mathrm{agg}}}=\sum_{s=1}^{S_{F}}\sigma_{s,o}^{t_{\mathrm{agg}}}$$

Due to a short distance between a small cell UE and its SBS and a low transmission power of an SBS, we assume similar indoor signal propagation characteristics for all *L* buildings per macrocell for an MNO *o* at *t*_agg_. Then, by linear approximation, the system-level average capacity of an MNO *o* at *t*_agg_ for *L* > 1 is given by,

(17) $$\sigma_{\mathrm{cap},o}^{\mathrm{sys},t_{\mathrm{agg}}}\left(L\right)=\sigma_{\mathrm{MBS},o}^{t_{\mathrm{agg}}}+\left(L\times \sigma_{S_{F},o}^{t_{\mathrm{agg}}}\right)$$

The SE for *L* buildings of an MNO *o* at *t*_agg_ is then given by,

(18) $$\sigma_{\mathrm{SE},o}^{\mathrm{sys},t_{\mathrm{agg}}}\left(L\right)=\sigma_{\mathrm{cap},o}^{\mathrm{sys},t_{\mathrm{agg}}}\left(L\right)/\left(\left(M_{\mathrm{MBS},o}+M_{o,t_{\mathrm{agg}}}\right)\times Q\right)$$

Let $P_{\mathrm{MC}}$, $P_{\mathrm{PC}}$, and $P_{\mathrm{SC}}$ denote, respectively, the transmission power of a macrocell, a picocell, and a small cell of an MNO *o.* The EE for *L* buildings of an MNO *o* at *t*_agg_ is then given by,

(19) $$\sigma_{\mathrm{EE},o}^{\mathrm{sys},t_{\mathrm{agg}}}\left(L\right)=\left(\begin{array}{l} \left(L\times S_{F}\times P_{\mathrm{SC}}\right)+\\ \left(S_{P}\times P_{\mathrm{PC}}\right)+\left(S_{M}\times P_{\mathrm{MC}}\right) \end{array}\right)/\left(\sigma_{\mathrm{cap},o}^{\mathrm{sys},t_{\mathrm{agg}}}\left(L\right)/Q\right)$$

#### 3.2.2. Case 2: All MNOs Countrywide

The system-level average capacity of all MNOs o∈{1,2,…,O} countrywide at *t*_agg_ for *L* > 1 is given by,

(20) $$\sigma_{\mathrm{cap},O}^{\mathrm{sys},t_{\mathrm{agg}}}\left(L\right)=\sum_{o=1}^{O}\sigma_{\mathrm{cap},o}^{\mathrm{sys},t_{\mathrm{agg}}}\left(L\right)$$

The SE for *L* buildings of all MNOs countrywide at *t*_agg_ is given by,

(21) $$\sigma_{\mathrm{SE},O}^{\mathrm{sys},t_{\mathrm{agg}}}\left(L\right)=\sigma_{\mathrm{cap},O}^{\mathrm{sys},t_{\mathrm{agg}}}\left(L\right)/\left(\sum_{o=1}^{O}\left(M_{\mathrm{MBS},o}+M_{o,t_{\mathrm{agg}}}\right)\times Q\right)$$

The EE for *L* buildings of all MNOs countrywide at *t*_agg_ is given by,

(22) $$\sigma_{\mathrm{EE},O}^{\mathrm{sys},t_{\mathrm{agg}}}\left(L\right)=\left(\begin{array}{l} \left(L\times S_{F}\times P_{\mathrm{SC}}\right)+\\ \left(S_{P}\times P_{\mathrm{PC}}\right)+\left(S_{M}\times P_{\mathrm{MC}}\right) \end{array}\right)/\left(\sigma_{\mathrm{cap},O}^{\mathrm{sys},t_{\mathrm{agg}}}\left(L\right)/Q\right)$$

### 3.3. Small Cell Network Performance

#### 3.3.1. Average Capacity

For the mmWave enabled small cells only, Equation (17) becomes as follows.

(23) $$\sigma_{\mathrm{cap},o}^{\mathrm{mmW},t_{\mathrm{agg}}}\left(L\right)=\left(L\times \sigma_{S_{F},o}^{t_{\mathrm{agg}}}\right)$$

Using Equation (23), the average aggregate capacity of the only mmWave enabled small cell networks for all MNOs at *t*_agg_ for *L*>1 is given by,

(24) $$\sigma_{\mathrm{cap},O}^{\mathrm{mmW},t_{\mathrm{agg}}}\left(L\right)=\sum_{o=1}^{O}\sigma_{\mathrm{cap},o}^{\mathrm{mmW},t_{\mathrm{agg}}}\left(L\right)$$

Let $\sigma_{\mathrm{cap},o,\mathrm{with}\;\mathrm{DESA}}^{\mathrm{mmW},t_{\mathrm{agg}}}\left(L\right)$ and $\sigma_{\mathrm{cap},o,\mathrm{without}\;\mathrm{DESA}}^{\mathrm{mmW},t_{\mathrm{agg}}}\left(L\right)$ denote, respectively, the average capacity for an MNO *o* at *t*_agg_ with applying and without applying DESA for mmWave enabled small cells only. Using Equation (23), $\sigma_{\mathrm{cap},o,\mathrm{with}\;\mathrm{DESA}}^{\mathrm{mmW},t_{\mathrm{agg}}}\left(L\right)$ and $\sigma_{\mathrm{cap},o,\mathrm{without}\;\mathrm{DESA}}^{\mathrm{mmW},t_{\mathrm{agg}}}\left(L\right)$ at *t*_agg_ for *L* > 1 can be expressed as follows.

(25) $$\sigma_{\mathrm{cap},o,\mathrm{with}\;\mathrm{DESA}}^{\mathrm{mmW},t_{\mathrm{agg}}}\left(L\right)=L\times \sum_{s=1}^{S_{F}}\sum_{t\in T}\sum_{i=1}^{M_{o,t_{\mathrm{agg}}}^{\mathrm{data}}}\sigma_{t,i,s,o}^{t_{\mathrm{agg}}}\left(\rho_{t,i,s,o}^{t_{\mathrm{agg}}}\right)$$

(26) $$\sigma_{\mathrm{cap},o,\mathrm{without}\;\mathrm{DESA}}^{\mathrm{mmW},t_{\mathrm{agg}}}\left(L\right)=L\times \sum_{s=1}^{S_{F}}\sum_{t\in T}\sum_{i=1}^{M_{o,t_{\mathrm{agg}}}^{\mathrm{data}}=\left(M-M_{o,t_{\mathrm{agg}}}^{\mathrm{res}}\right)}\sigma_{t,i,s,o}^{t_{\mathrm{agg}}}\left(\rho_{t,i,s,o}^{t_{\mathrm{agg}}}\right)$$

Let $\sigma_{\mathrm{cap},O,\mathrm{with}\;\mathrm{DESA}}^{\mathrm{mmW},t_{\mathrm{agg}}}\left(L\right)$ and $\sigma_{\mathrm{cap},O,\mathrm{without}\;\mathrm{DESA}}^{\mathrm{mmW},t_{\mathrm{agg}}}\left(L\right)$ denote, respectively, the countrywide average capacity at *t*_agg_ with applying and without applying DESA, which can be expressed as follows.

(27) $$\sigma_{\mathrm{cap},O,\mathrm{with}\;\mathrm{DESA}}^{\mathrm{mmW},t_{\mathrm{agg}}}\left(L\right)=\sum_{o=1}^{O}\left(L\times \sum_{s=1}^{S_{F}}\sum_{t\in T}\sum_{i=1}^{M_{o,t_{\mathrm{agg}}}^{\mathrm{data}}}\sigma_{t,i,s,o}^{t_{\mathrm{agg}}}\left(\rho_{t,i,s,o}^{t_{\mathrm{agg}}}\right)\right)$$

(28) $$\sigma_{\mathrm{cap},O,\mathrm{without}\;\mathrm{DESA}}^{\mathrm{mmW},t_{\mathrm{agg}}}\left(L\right)=\sum_{o=1}^{O}\left(L\times \sum_{s=1}^{S_{F}}\sum_{t\in T}\sum_{i=1}^{M_{o,t_{\mathrm{agg}}}^{\mathrm{data}}=\left(M-M_{o,t_{\mathrm{agg}}}^{\mathrm{res}}\right)}\sigma_{t,i,s,o}^{t_{\mathrm{agg}}}\left(\rho_{t,i,s,o}^{t_{\mathrm{agg}}}\right)\right)$$

Hence, the factor representing an improvement in average capacity due to applying DESA can be expressed, respectively, for an MNO *o* and all MNOs countrywide at term *t*_agg_ as follows.

(29) $$\varsigma_{\mathrm{cap},o,\mathrm{IF}}^{\mathrm{mmW},t_{\mathrm{agg}}}\left(L\right)=\sigma_{\mathrm{cap},o,\mathrm{with}\;\mathrm{DESA}}^{\mathrm{mmW},t_{\mathrm{agg}}}\left(L\right)/\sigma_{\mathrm{cap},o,\mathrm{without}\;\mathrm{DESA}}^{\mathrm{mmW},t_{\mathrm{agg}}}\left(L\right)$$

(30) $$\varsigma_{\mathrm{cap},O,\mathrm{IF}}^{\mathrm{mmW},t_{\mathrm{agg}}}\left(L\right)=\sigma_{\mathrm{cap},O,\mathrm{with}\;\mathrm{DESA}}^{\mathrm{mmW},t_{\mathrm{agg}}}\left(L\right)/\sigma_{\mathrm{cap},O,\mathrm{without}\;\mathrm{DESA}}^{\mathrm{mmW},t_{\mathrm{agg}}}\left(L\right)$$

#### 3.3.2. Spectral Efficiency

The available spectrum at any term *t*_agg_ for an MNO *o* without applying DESA does not change and is given by $M_{o,t_{\mathrm{agg}}}=M_{o,t_{\mathrm{agg}}=0}=M$. However, with applying DESA, the available spectrum for an MNO *o* is given by $M_{o,t_{\mathrm{agg}}}=\left(M_{o,t_{\mathrm{agg}}}^{\mathrm{data}}+M_{o,t_{\mathrm{agg}}}^{\mathrm{res}}\right)$. Let $\sigma_{\mathrm{SE},o,\mathrm{with}\;\mathrm{DESA}}^{\mathrm{mmW},t_{\mathrm{agg}}}\left(L\right)$ and $\sigma_{\mathrm{SE},o,\mathrm{without}\;\mathrm{DESA}}^{\mathrm{mmW},t_{\mathrm{agg}}}\left(L\right)$ denote, respectively, the SE for an MNO *o* at *t*_agg_ with applying and without applying DESA, which are given by,

(31) $$\sigma_{\mathrm{SE},o,\mathrm{with}\;\mathrm{DESA}}^{\mathrm{mmW},t_{\mathrm{agg}}}\left(L\right)=\sigma_{\mathrm{cap},o,\mathrm{with}\;\mathrm{DESA}}^{\mathrm{mmW},t_{\mathrm{agg}}}\left(L\right)/\left(\left(M_{o,t_{\mathrm{agg}}}^{\mathrm{data}}+M_{o,t_{\mathrm{agg}}}^{\mathrm{res}}\right)\times Q\right)$$

(32) $$\sigma_{\mathrm{SE},o,\mathrm{without}\;\mathrm{DESA}}^{\mathrm{mmW},t_{\mathrm{agg}}}\left(L\right)=\sigma_{\mathrm{cap},o,\mathrm{without}\;\mathrm{DESA}}^{\mathrm{mmW},t_{\mathrm{agg}}}\left(L\right)/\left(M\times Q\right)$$

Likewise, let $\sigma_{\mathrm{SE},O,\mathrm{with}\;\mathrm{DESA}}^{\mathrm{mmW},t_{\mathrm{agg}}}\left(L\right)$ and $\sigma_{\mathrm{SE},O,\mathrm{without}\;\mathrm{DESA}}^{\mathrm{mmW},t_{\mathrm{agg}}}\left(L\right)$ denote, respectively, the countrywide SE at *t*_agg_ with applying and without applying DESA, which can be expressed as follows.

(33) $$\sigma_{\mathrm{SE},O,\mathrm{with}\;\mathrm{DESA}}^{\mathrm{mmW},t_{\mathrm{agg}}}\left(L\right)=\sigma_{\mathrm{cap},O,\mathrm{with}\;\mathrm{DESA}}^{\mathrm{mmW},t_{\mathrm{agg}}}\left(L\right)/\left(\left(\sum_{o=1}^{O}\left(M_{o,t_{\mathrm{agg}}}^{\mathrm{data}}+M_{o,t_{\mathrm{agg}}}^{\mathrm{res}}\right)\right)\times Q\right)$$

(34) $$\sigma_{\mathrm{SE},O,\mathrm{without}\;\mathrm{DESA}}^{\mathrm{mmW},t_{\mathrm{agg}}}\left(L\right)=\sigma_{\mathrm{cap},O,\mathrm{without}\;\mathrm{DESA}}^{\mathrm{mmW},t_{\mathrm{agg}}}\left(L\right)/\left(\left(O\times M\right)\times Q\right)$$

Hence, the factor representing an improvement in SE due to applying DESA can be expressed, respectively, for an MNO *o* and all MNOs countrywide as follows.

(35) $$\varsigma_{\mathrm{SE},o,\mathrm{IF}}^{\mathrm{mmW},t_{\mathrm{agg}}}\left(L\right)=\sigma_{\mathrm{SE},o,\mathrm{with}\;\mathrm{DESA}}^{\mathrm{mmW},t_{\mathrm{agg}}}\left(L\right)/\sigma_{\mathrm{SE},o,\mathrm{without}\;\mathrm{DESA}}^{\mathrm{mmW},t_{\mathrm{agg}}}\left(L\right)$$

(36) $$\varsigma_{\mathrm{SE},O,\mathrm{IF}}^{\mathrm{mmW},t_{\mathrm{agg}}}\left(L\right)=\sigma_{\mathrm{SE},O,\mathrm{with}\;\mathrm{DESA}}^{\mathrm{mmW},t_{\mathrm{agg}}}\left(L\right)/\sigma_{\mathrm{SE},O,\mathrm{without}\;\mathrm{DESA}}^{\mathrm{mmW},t_{\mathrm{agg}}}\left(L\right)$$

#### 3.3.3. Energy Efficiency

Let $\sigma_{\mathrm{EE},o,\mathrm{with}\;\mathrm{DESA}}^{\mathrm{mmW},t_{\mathrm{agg}}}\left(L\right)$ and $\sigma_{\mathrm{EE},o,\mathrm{without}\;\mathrm{DESA}}^{\mathrm{mmW},t_{\mathrm{agg}}}\left(L\right)$ denote, respectively, the EE for an MNO *o* at *t*_agg_ with applying and without applying DESA, which can be expressed as follows.

(37) $$\sigma_{\mathrm{EE},o,\mathrm{with}\;\mathrm{DESA}}^{\mathrm{mmW},t_{\mathrm{agg}}}\left(L\right)=\left(\begin{array}{l} \left(L\times S_{F}\times P_{\mathrm{SC}}\right)+\\ \left(S_{P}\times P_{\mathrm{PC}}\right)+\left(S_{M}\times P_{\mathrm{MC}}\right) \end{array}\right)/\left(\sigma_{\mathrm{cap},o,\mathrm{with}\;\mathrm{DESA}}^{\mathrm{mmW},t_{\mathrm{agg}}}\left(L\right)/Q\right)$$

(38) $$\sigma_{\mathrm{EE},o,\mathrm{without}\;\mathrm{DESA}}^{\mathrm{mmW},t_{\mathrm{agg}}}\left(L\right)=\left(\begin{array}{l} \left(L\times S_{F}\times P_{\mathrm{SC}}\right)+\\ \left(S_{P}\times P_{\mathrm{PC}}\right)+\left(S_{M}\times P_{\mathrm{MC}}\right) \end{array}\right)/\left(\sigma_{\mathrm{cap},o,\mathrm{without}\;\mathrm{DESA}}^{\mathrm{mmW},t_{\mathrm{agg}}}\left(L\right)/Q\right)$$

Similarly, let $\sigma_{\mathrm{EE},O,\mathrm{with}\;\mathrm{DESA}}^{\mathrm{mmW},t_{\mathrm{agg}}}\left(L\right)$ and $\sigma_{\mathrm{EE},O,\mathrm{without}\;\mathrm{DESA}}^{\mathrm{mmW},t_{\mathrm{agg}}}\left(L\right)$ denote, respectively, the countrywide EE at *t*_agg_ with applying and without applying DESA, which can be expressed as follows.

(39) $$\sigma_{\mathrm{EE},O,\mathrm{with}\;\mathrm{DESA}}^{\mathrm{mmW},t_{\mathrm{agg}}}\left(L\right)=\left(O\times \left(\begin{array}{l} \left(L\times S_{F}\times P_{\mathrm{SC}}\right)+\\ \left(S_{P}\times P_{\mathrm{PC}}\right)+\left(S_{M}\times P_{\mathrm{MC}}\right) \end{array}\right)\right)/\left(\sigma_{\mathrm{cap},O,\mathrm{with}\;\mathrm{DESA}}^{\mathrm{mmW},t_{\mathrm{agg}}}\left(L\right)/Q\right)$$

(40) $$\sigma_{\mathrm{EE},O,\mathrm{without}\;\mathrm{DESA}}^{\mathrm{mmW},t_{\mathrm{agg}}}\left(L\right)=\left(O\times \left(\begin{array}{l} \left(L\times S_{F}\times P_{\mathrm{SC}}\right)+\\ \left(S_{P}\times P_{\mathrm{PC}}\right)+\left(S_{M}\times P_{\mathrm{MC}}\right) \end{array}\right)\right)/\left(\sigma_{\mathrm{cap},O,\mathrm{without}\;\mathrm{DESA}}^{\mathrm{mmW},t_{\mathrm{agg}}}\left(L\right)/Q\right)$$

Like SE, the factor representing an improvement in EE due to applying DESA can be expressed, respectively, for an MNO *o* and all MNOs countrywide as follows.

(41) $$\varsigma_{\mathrm{EE},o,\mathrm{IF}}^{\mathrm{mmW},t_{\mathrm{agg}}}\left(L\right)=\sigma_{\mathrm{EE},o,\mathrm{with}\;\mathrm{DESA}}^{\mathrm{mmW},t_{\mathrm{agg}}}\left(L\right)/\sigma_{\mathrm{EE},o,\mathrm{without}\;\mathrm{DESA}}^{\mathrm{mmW},t_{\mathrm{agg}}}\left(L\right)$$

(42) $$\varsigma_{\mathrm{EE},O,\mathrm{IF}}^{\mathrm{mmW},t_{\mathrm{agg}}}\left(L\right)=\sigma_{\mathrm{EE},O,\mathrm{with}\;\mathrm{DESA}}^{\mathrm{mmW},t_{\mathrm{agg}}}\left(L\right)/\sigma_{\mathrm{EE},O,\mathrm{without}\;\mathrm{DESA}}^{\mathrm{mmW},t_{\mathrm{agg}}}\left(L\right)$$

#### 3.3.4. Cost Efficiency

Let $\varepsilon_{C}$ denote the cost of the total amount of mmWave spectrum $M_{C,\max}$ allocated to a country where an MNO *o* pays $\varepsilon_{o}$ for its licensed spectrum $M_{o,t_{\mathrm{agg}}=0}$ such that $\varepsilon_{C}$ can be expressed as follows.

(43) $$\varepsilon_{C}=\sum_{o=1}^{O}\varepsilon_{o}$$

where $\varepsilon_{o}$ and $\varepsilon_{C}$ are expressed in per Hertz of the licensed spectrum and fixed for a long time.

Using (23)–(24), defining the cost efficiency (CE) as the cost required per unit achievable average capacity (i.e., bps), we can find the CE of small cell networks for *L* > 1 at *t*_agg_, respectively, for an MNO *o* and all MNOs countrywide as follows.

(44) $$\varsigma_{\mathrm{CE},o}^{\mathrm{mmW},t_{\mathrm{agg}}}\left(L\right)=\varepsilon_{o}/\sigma_{\mathrm{cap},o}^{\mathrm{mmW},t_{\mathrm{agg}}}\left(L\right)$$

(45) $$\varsigma_{\mathrm{CE},O}^{\mathrm{mmW},t_{\mathrm{agg}}}\left(L\right)=\varepsilon_{C}/\sigma_{\mathrm{cap},O}^{\mathrm{mmW},t_{\mathrm{agg}}}\left(L\right)$$

Let $\varsigma_{\mathrm{CE},o,\mathrm{with}\;\mathrm{DESA}}^{\mathrm{mmW},t_{\mathrm{agg}}}\left(L\right)$ and $\varsigma_{\mathrm{CE},o,\mathrm{without}\;\mathrm{DESA}}^{\mathrm{mmW},t_{\mathrm{agg}}}\left(L\right)$ denote, respectively, the CE of an MNO *o* at *t*_agg_ with applying and without applying DESA, which can be expressed as follows.

(46) $$\varsigma_{\mathrm{CE},o,\mathrm{with}\;\mathrm{DESA}}^{\mathrm{mmW},t_{\mathrm{agg}}}\left(L\right)=\varepsilon_{o}/\left(L\times \sum_{s=1}^{S_{F}}\sum_{t\in T}\sum_{i=1}^{M_{o,t_{\mathrm{agg}}}^{\mathrm{data}}}\sigma_{t,i,s,o}^{t_{\mathrm{agg}}}\left(\rho_{t,i,s,o}^{t_{\mathrm{agg}}}\right)\right)$$

(47) $$\varsigma_{\mathrm{CE},o,\mathrm{without}\;\mathrm{DESA}}^{\mathrm{mmW},t_{\mathrm{agg}}}\left(L\right)=\varepsilon_{o}/\left(L\times \sum_{s=1}^{S_{F}}\sum_{t\in T}\sum_{i=1}^{M_{o,t_{\mathrm{agg}}}^{\mathrm{data}}=\left(M-M_{o,t_{\mathrm{agg}}}^{\mathrm{res}}\right)}\sigma_{t,i,s,o}^{t_{\mathrm{agg}}}\left(\rho_{t,i,s,o}^{t_{\mathrm{agg}}}\right)\right)$$

Similarly, let $\varsigma_{\mathrm{CE},O,\mathrm{with}\;\mathrm{DESA}}^{\mathrm{mmW},t_{\mathrm{agg}}}\left(L\right)$ and $\varsigma_{\mathrm{CE},O,\mathrm{without}\;\mathrm{DESA}}^{\mathrm{mmW},t_{\mathrm{agg}}}\left(L\right)$ denote, respectively, the countrywide CE at *t*_agg_ with applying and without applying DESA, which can be expressed as follows.

(48) $$\varsigma_{\mathrm{CE},O,\mathrm{with}\;\mathrm{DESA}}^{\mathrm{mmW},t_{\mathrm{agg}}}\left(L\right)=\varepsilon_{C}/\sum_{o=1}^{O}\left(L\times \sum_{s=1}^{S_{F}}\sum_{t\in T}\sum_{i=1}^{M_{o,t_{\mathrm{agg}}}^{\mathrm{data}}}\sigma_{t,i,s,o}^{t_{\mathrm{agg}}}\left(\rho_{t,i,s,o}^{t_{\mathrm{agg}}}\right)\right)$$

(49) $$\varsigma_{\mathrm{CE},O,\mathrm{without}\;\mathrm{DESA}}^{\mathrm{mmW},t_{\mathrm{agg}}}\left(L\right)=\varepsilon_{C}/\sum_{o=1}^{O}\left(L\times \sum_{s=1}^{S_{F}}\sum_{t\in T}\sum_{i=1}^{M_{o,t_{\mathrm{agg}}}^{\mathrm{data}}=\left(M-M_{o,t_{\mathrm{agg}}}^{\mathrm{res}}\right)}\sigma_{t,i,s,o}^{t_{\mathrm{agg}}}\left(\rho_{t,i,s,o}^{t_{\mathrm{agg}}}\right)\right)$$

Hence, the factor representing an improvement in CE due to applying DESA can be expressed, respectively, for an MNO *o* and all MNOs countrywide as follows.

(50) $$\varsigma_{\mathrm{CE},o,\mathrm{IF}}^{\mathrm{mmW},t_{\mathrm{agg}}}\left(L\right)=\varsigma_{\mathrm{CE},o,\mathrm{with}\;\mathrm{DESA}}^{\mathrm{mmW},t_{\mathrm{agg}}}\left(L\right)/\varsigma_{\mathrm{CE},o,\mathrm{without}\;\mathrm{DESA}}^{\mathrm{mmW},t_{\mathrm{agg}}}\left(L\right)$$

(51) $$\varsigma_{\mathrm{CE},O,\mathrm{IF}}^{\mathrm{mmW},t_{\mathrm{agg}}}\left(L\right)=\varsigma_{\mathrm{CE},O,\mathrm{with}\;\mathrm{DESA}}^{\mathrm{mmW},t_{\mathrm{agg}}}\left(L\right)/\varsigma_{\mathrm{CE},O,\mathrm{without}\;\mathrm{DESA}}^{\mathrm{mmW},t_{\mathrm{agg}}}\left(L\right)$$

## 4. Performance Evaluation

### 4.1. Default Parameter and Assumption

Table 1 shows default simulation parameters and assumptions considered for the performance evaluation of the proposed DESA method. Default simulation assumptions, parameters, and models used for the performance evaluation are in line with the recommendations from the standardization bodies such as the third-generation partnership project (3GPP) and International Telecommunication Union-Radiocommunication Sector (ITU-R). Besides, performance results were generated simulating all assumptions, parameters, and models given in Table 1 by a simulator built using the computational tool MATLAB R2012b version running on a personal computer. Given in Table 1, as one of the effective mmWave bands for 5G, as well as the future 6G [34], mobile systems, the 28 GHz band is considered for the evaluation of mmWave mobile systems. Moreover, even though the 28 GHz mmWave spectrum allocated to an MNO in a country can be large enough (e.g., 400 MHz [35]), for simplicity, we consider that each MNO of a country is allocated to an equal amount of spectrum of 50 GHz. 

Likewise, the number of MNOs operating in a country is considered four. However, the proposed DESA method is generic, which can be applied to evaluate any number of MNOs in a country each assigned unevenly with a dedicated spectrum bandwidth of other mmWave bands as well. Further, due to less multipath fading effect of high-frequency signals in indoor environments, we consider the line-of-sight (LOS) large-scale path loss model for the 28 GHz mmWave signals within buildings. Furthermore, because of a small coverage and less multipath fading effect of an indoor small cell operating at the 28 GHz band, we assume a similar mmWave signal propagation characteristic within each building. Besides, due to the high external wall penetration loss of a building, the same 28 GHz mmWave spectrum can be reused to small cells located in adjacent buildings to evaluate the proposed DESA method with a view to addressing the SE and EE requirements for 6G mobile systems. Finally, because of providing balance performances between throughputs and fairness in radio resource allocations, the proportional fair (PF) resource scheduler is considered. Further, the full buffer model is considered for simplicity such that resource schedulers can be assumed to have user traffic to serve at any time over the observation period *Q*.

### 4.2. Performance Result

#### 4.2.1. Shared Spectrum per MNO

Assume that each MNO in a country is allocated statically to an equal amount of 50 MHz mmWave spectrum irrespective of the number of their subscribers. In addition, each MNO considers reserving 20% of its allocated spectrum (i.e., 10 MHz) for control signaling and other system-specific requirements. Hence, without applying DESA, the available 28 GHz mmWave spectrum for serving the data traffic of each MNO is given by 40 MHz. Now, applying DESA and using Equation (10) and Table 1, we can find the optimal amount of shared spectrum to serve data traffic for MNO 1 at *t*_agg_ is given as follows.

$$M_{1,t_{\mathrm{agg}}}^{\mathrm{shared}}{\;}^{*}=\;\left(\frac{0.4N_{C,\max}\times \left(4\times \left(50-10\right)\mathrm{MHz}\right)}{\left(0.4N_{C,\max}+0.3N_{C,\max}+0.2N_{C,\max}+0.1N_{C,\max}\right)}\right)-\left(50-10\right)\mathrm{MHz}$$

$$\begin{array}{l} M_{1,t_{\mathrm{agg}}}^{\mathrm{shared}}{\;}^{*}=\left(\frac{\left(0.4\times 160\;\mathrm{MHz}\right)}{1}-40\;\mathrm{MHz}\right)\\ M_{1,t_{\mathrm{agg}}}^{\mathrm{shared}}{\;}^{*}=+24\;\mathrm{MHz} \end{array}$$

**Figure 4:**
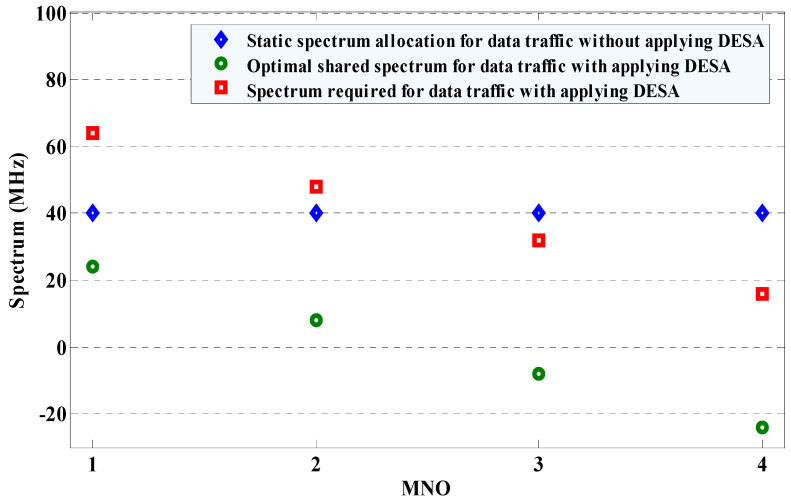
The spectrum redistribution to all MNOs in a country with applying DESA.

Following the above procedure, we can find as well the optimal amount of shared spectrum for other MNOs as +8 MHz for MNO 2, whereas -8 and -24 MHz for MNOs 3 and 4, respectively, as shown in Figure 4. As mentioned above, these optimal amounts of shared spectra for all MNOs can be justified by the fact that $\sum_{o=1}^{O}M_{o,t_{\mathrm{agg}}}^{\mathrm{shared}}{\;}^{*}=\left(+24+8-8-24\right)=0$. Recall that an MNO with a positive sign in the shared spectrum implies that the MNO has a shortage of the shared spectrum, whereas a negative sign implies that an MNO has an excessive of the shared spectrum. Hence, since MNOs 3 and 4 have an excess of spectra of 8 and 24 MHz, respectively, even after serving their user demands, MNOs 1 and 2 can lease their shared spectra of 24 and 8 GHz from MNOs 4 and 3, respectively, to use exclusively at the cost of payment for the duration of the agreement term *t*_agg_. Therefore, the spectra owned by MNOs 1, 2, 3, and 4 after sharing the spectrum of MNO 4 with MNO 1 and MNO 3 with MNO 2, are given, respectively, by 64, 48, 32, and 16 MHz as shown in Figure 4 to serve uniformly data traffic demands of all subscribers in the country.

#### 4.2.2. Performance Metrics of all MNOs

**Figure 5:**
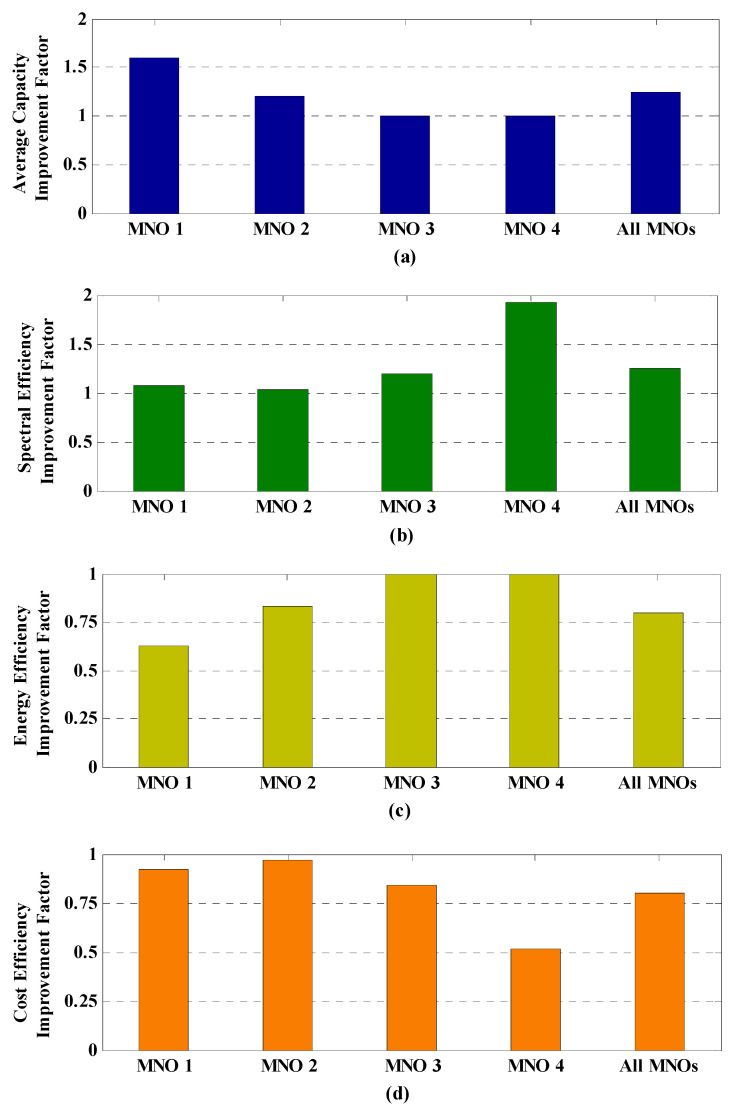
Average capacity, SE, EE, and CE improvement factors of all MNOs due to applying DESA for *L* = 1: (**a**) average capacity; (**b**) SE; (**c**) EE; and (**d**) CE.

Figure 5 shows the average capacity, SE, EE, and CE performances for each MNO (using Equations (29), (35), (41), and (50), respectively), as well as for all MNOs countrywide (using Equations (30), (36), (42), and (51), respectively), for a single building of small cells (i.e., for *L* = 1). Figure 5a shows that, applying the proposed DESA method, the average capacity of MNOs 1 and 2 is increased due to the additional spectra of $\left|M_{1,t_{\mathrm{agg}}}^{\mathrm{shared}}{\;}^{*}\right|$ and $\left|M_{2,t_{\mathrm{agg}}}^{\mathrm{shared}}{\;}^{*}\right|$, respectively, at *t*_agg_, which are obtained by leasing from MNOs 4 and 3, respectively, without affecting the average capacities required by MNOs 4 and 3. This can be clarified by the fact that MNOs 3 and 4 have a significant amount of unused spectra given by $\left|M_{3,t_{\mathrm{agg}}}^{\mathrm{shared}}{\;}^{*}\right|$ and $\left|M_{4,t_{\mathrm{agg}}}^{\mathrm{shared}}{\;}^{*}\right|$, respectively, such that, without applying DESA, the mmWave spectrum allocated statically to each MNO is not utilized fully resulting in poor average capacity responses for all MNOs countrywide. However, with applying DESA, MNOs 3 and 4 keep only the amount of spectrum that is necessary to satisfy their respective data traffic demand of users, while the remaining amount of spectra given by $\left|M_{4,t_{\mathrm{agg}}}^{\mathrm{shared}}{\;}^{*}\right|$ for MNO 4 and $\left|M_{3,t_{\mathrm{agg}}}^{\mathrm{shared}}{\;}^{*}\right|$ for MNO 3 is leased to MNOs 1 and 2, respectively, to gain additional profit. Because of utilizing the unused spectra of MNOs 4 and 3 by MNOs 1 and 2, respectively, the total achievable capacity of all MNOs countrywide is improved by 25% at *t*_agg_ using the same spectrum allocated to the country resulting in serving more data traffic countrywide with applying DESA, as shown in Figure 5a.

However, Figure 5b shows that seller MNOs 3 and 4 provide better performance in SE with applying DESA than buyer MNOs 1 and 2. The SE of MNOs 3 and 4 increase with applying DESA due to the proper utilization of their unused spectra $\left|M_{3,t_{\mathrm{agg}}}^{\mathrm{shared}}{\;}^{*}\right|$ and $\left|M_{4,t_{\mathrm{agg}}}^{\mathrm{shared}}{\;}^{*}\right|$ by leasing them to MNOs 2 and 1, respectively (Figure 5b). Particularly, a seller MNO with the least number of subscribers provides the best SE response. Since MNOs 1 and 2 can make full utilization of their respective spectra $\left|M_{1,t_{\mathrm{agg}}}^{\mathrm{data}}\right|$ and $\left|M_{2,t_{\mathrm{agg}}}^{\mathrm{data}}\right|$ by leasing their respective lack of spectra $\left|M_{1,t_{\mathrm{agg}}}^{\mathrm{shared}}{\;}^{*}\right|$ and $\left|M_{2,t_{\mathrm{agg}}}^{\mathrm{shared}}{\;}^{*}\right|$, the SE of MNOs 1 and 2 do not increase noticeably, as shown in Figure 5b. Note that the improvement in average capacity and SE of an MNO with applying DESA method depends on the amount of leased spectrum. In general, an MNO with a positive sign that requires more shared spectrum results in a corresponding higher average capacity response with applying DESA. Similarly, an MNO with a negative sign that requires more shared spectrum results in a corresponding higher SE response with applying DESA. This is reflected in Figure 5a,b, which shows that MNOs 1 and 4 can achieve higher average capacity and SE responses than MNOs 2 and 3, respectively.

Figure 5c shows the EE responses of all MNOs. Since the total amount of transmission power is the same for each MNO irrespective of applying DESA, the EE response of an MNO depends directly on its average capacity response shown in Figure 5a. Recall that the EE (i.e., the energy required per bit transmission) is inversely related to the average capacity for an MNO (as given by Equations (37) and (38)). Hence, using Figure 5a, since the average capacity of MNO 1 with applying DESA is higher than that of MNO 2, the energy required per bit transmission for MNO 1 is also lower than that of MNO 2 (Figure 5c). More specifically, with applying DESA, an improvement in EE of 37.5% and 17.5%, respectively, for MNOs 1 and 2 can be obtained. Accordingly, no improvement in the EE performance is observed for MNOs 3 and 4 with applying DESA, which is shown in Figure 5c by the improvement factor of unity for MNOs 3 and 4. However, similar to the average capacity response, the countrywide EE response is also improved by 17.5% with applying DESA.

Finally, Figure 5d shows the CE (i.e., the cost required per unit average capacity) responses of all MNOs where it can be found that MNOs 3 and 4 provide better CE performances due to gaining additional profits from leasing their unused spectra such that the cost required per bit per second (bps) (i.e., per unit average capacity) is lower than that of MNOs 1 and 2. More specifically, since MNO 1 pays the highest amount of money for its leased spectrum to MNO 4, followed by MNO 2 for its leased spectrum to MNO 3 at *t*_agg_, the cost required per bps of MNO 4 is the lowest followed by that of MNO 3, as shown in Figure 5d. Accordingly, even though MNOs 1 and 2 can address their respective user demands by achieving more capacity with applying DESA, they have to pay the cost for their leased spectra to MNOs 4 and 3, respectively, resulting in increasing the cost for serving per unit bps for MNOs 1 and 2 (Figure 5d). However, similar to the average capacity, the countrywide CE is also improved by 20% with applying DESA.

In short, only with applying DESA, to address the increased average capacity demands of users, both MNOs 1 and 2 can lease additional spectra of $\left|M_{1,t_{\mathrm{agg}}}^{\mathrm{shared}}{\;}^{*}\right|$ and $\left|M_{2,t_{\mathrm{agg}}}^{\mathrm{shared}}{\;}^{*}\right|$ from MNOs 4 and 3, respectively, at the cost of paying for their leased spectra to MNOs 4 and 3 at *t*_agg_, who would otherwise under-utilize their spectra given by $\left|M_{4,t_{\mathrm{agg}}}^{\mathrm{shared}}{\;}^{*}\right|$ and $\left|M_{3,t_{\mathrm{agg}}}^{\mathrm{shared}}{\;}^{*}\right|$. Such a kind of spectrum trading results in the increased EE of MNOs 1 and 2 as well, whereas the increased SE and CE of MNOs 4 and 3. In other words, the proposed DESA method makes a balance by redistributing the total countrywide spectra among MNOs 1, 2, 3, and 4 at *t*_agg_ such that the overall countrywide average capacity, SE, EE, and CE can be improved as shown in Figure 5.

**Figure 6:**
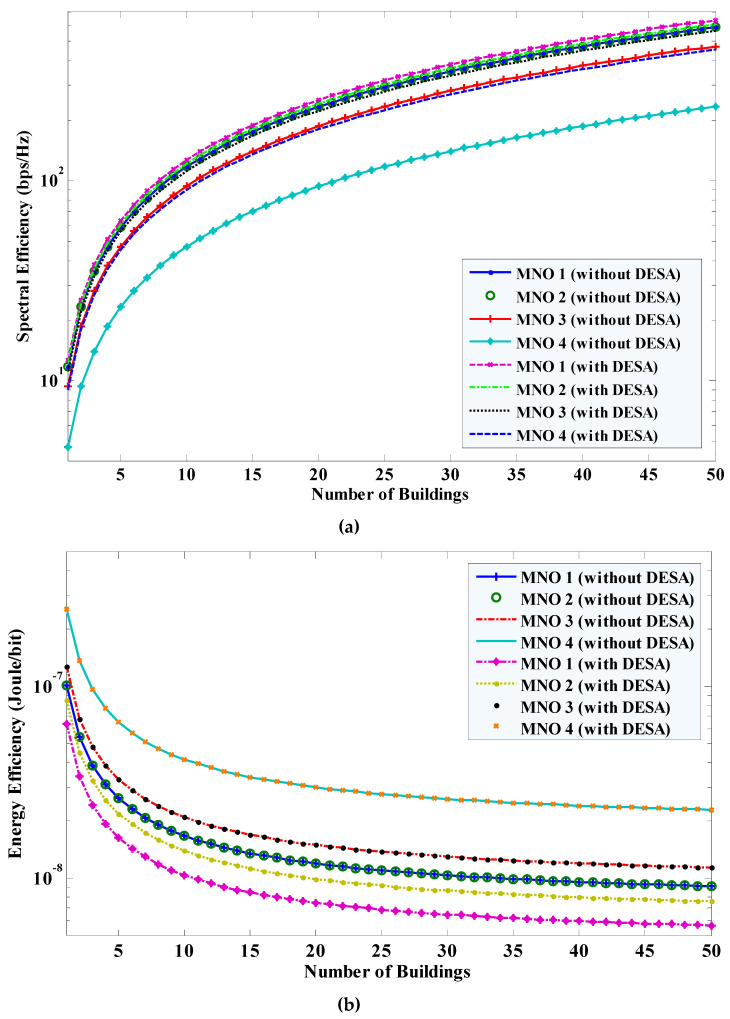
SE and EE performances of all MNOs with applying and without applying DESA for *L*>1: (**a**) SE; and (**b**) EE.

Moreover, by extending the results shown in Figure 5b,c for *L* = 1, the SE (using Equations (31) and (32)) and EE (using Equations (37) and (38)) performances of all MNOs when reusing the mmWave spectrum of each MNO to *L* number of buildings of small cells are shown in Figure 6a,b, respectively. In Figure 6, it can be found that the SE of all MNOs increases linearly with an increase in the number of buildings of small cells *L*. However, the EE of all MNOs improves negative exponentially and gets almost fixed as *L* tends to a large number.

## 5. Performance Comparison and Case Study

### 5.1. Performance Comparison

According to Zhang, Z. *et al.* [42], the future 6G mobile systems are expected to require 10 times average SE (i.e., 270–370 bps/Hz), as well as 10 times average EE [34] (i.e., 0.3×10^−6^ Joules/bit), of 5G mobile systems [43,44]. Denote $\sigma_{\mathrm{SE}}^{6G}$ and $\sigma_{\mathrm{EE}}^{6G}$, respectively, as the average SE and average EE requirements for 6G mobile systems such that $\sigma_{\mathrm{SE}}^{6G}$ = 370 bps/Hz and $\sigma_{\mathrm{EE}}^{6G}$ = 0.3 µJ/b. Using Figure 6, the number of buildings of small cells *L* required for each MNO with applying, as well as without applying, DESA is given in Table 2.

In Table 2, it can be found that the value of *L* is strictly defined by the SE requirement for 6G mobile systems. Because of leasing the unused spectra from MNOs 4 and 3 by MNOs 1 and 2, respectively, MNO 4 requires the minimum number of buildings of small cells, followed by MNO 3, when reusing the 28 GHz mmWave spectrum to each building to achieve the expected SE and EE requirements for 6G mobile systems. Further, since MNOs 1 and 2 both operate at their respective maximum available spectra irrespective of applying DESA, the required value of *L* with applying DESA does not change noticeably from that required without applying DESA for MNOs 1 and 2.

**Figure 7:**
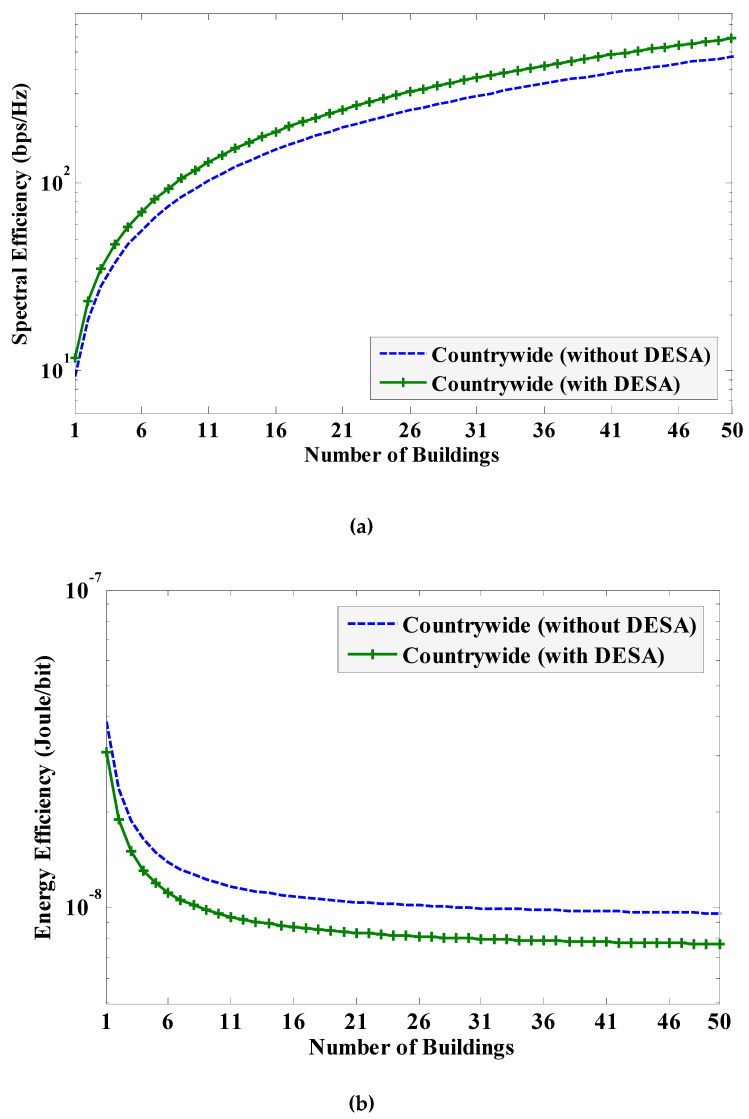
Countrywide SE and EE responses with the variation of the number of buildings of small cells *L* per MNO: (**a**) SE; and (**b**) EE.

Now, using Figure 7, the countrywide average SE and average EE responses due to reusing the same mmWave spectrum to *L* buildings of small cells per MNO are also given in Table 2. In Table 2, it can be found that, without applying DESA to all MNOs countrywide, the number of buildings of small cells per MNO required to achieve the SE and EE requirements for 6G mobile systems is 40. However, when applying DESA to all MNOs countrywide, the required number of buildings is reduced (by 20% of that required without applying DESA) to 32. Hence, by applying DESA, the expected SE and EE requirements for 6G mobile systems can be achieved by reusing the same 28 GHz mmWave spectrum to 47.5%, 15%, and 20% fewer buildings of small cells, respectively, for MNO 4, MNO 3, and all MNOs countrywide.

### 5.2. Case Study-Applying DESA in the Perspective of Four MNOs in a Country for 5G

**Figure 8:**
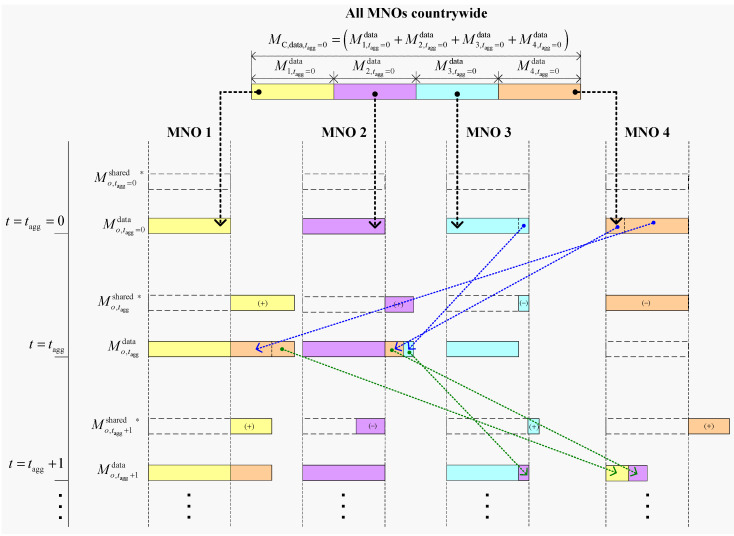
An illustration of the application of DESA to an arbitrary country of four MNOs for 5G services with the 28 GHz mmWave spectrum.

The proposed DESA method has a substantial impact on MNOs with diverse traffic demands in a country. To demonstrate this, we consider applying DESA to an arbitrary country with four MNOs, namely MNO 1, MNO 2, MNO 3, and MNO 4 as a case study for 5G networks in the following. Assume that each MNO is granted statically with the same amount of 28 GHz mmWave spectrum of 400 MHz for 5G mobile services, i.e., $\forall o\;M_{o,t_{\mathrm{agg}}=0}=M=400\;\mathrm{MHz}$, irrespective of the number of subscribers per MNO. Figure 8 shows the application of the proposed DESA method to all four MNOs to update the shared spectrum of each MNO, i.e., $\forall oM_{o,t_{\mathrm{agg}}}^{\mathrm{shared}}{\;}^{*}$, at consecutive agreement terms using the iterative algorithm. Assume that the spectrum required by an MNO is proportional to its number of existing subscribers such that MNO 1 serves 44%, MNO 2 serves 32%, MNO 3 serves 24%, and MNO 4 serves 0%. Hence, with respect to the largest number of subscribers of MNO 1, the relative unit values of MNO 1, MNO 2, MNO 3, and MNO 4 are, respectively, 1, 0.75, 0.55, 0.

Now, assume that each MNO reserves about 25% of its allocated mmWave spectrum for control signaling, coordination, emergency, and other system-specific requirements. Hence, the reserved spectrum per MNO equals 100 MHz i.e., $\forall oM_{o,t_{\mathrm{agg}}}^{\mathrm{reserved}}=100\;\mathrm{MHz}\;$ since each MNO is assigned with a dedicated mmWave spectrum of 400 MHz in the 28 GHz band. This implies that a total of 300 MHz is available for each MNO to serve its users’ data traffic, i.e., $\forall oM_{o,t_{\mathrm{agg}}=0}^{\mathrm{data}}=300\;\mathrm{MHz}$. Therefore, the effective spectrum of 1200 MHz (i.e., 300 × 4 MHz) in the 28 GHz band is available to serve the total data traffic of four MNOs countrywide, i.e., $M_{C,\mathrm{data},t_{\mathrm{agg}}=0}=1200\;\mathrm{MHz}$, as shown in Figure 8. Now, using the ratio of the unit value for each MNO with respect to the sum of the total unit values given above for each MNO (i.e., 1, 0.75, 0.55, and 0), the spectrum required for serving data traffic of one MNO varies from another, particularly 533.3 MHz for MNO 1, 400 MHz for MNO 2, 266.67 MHz MNO 3, and 0 MHz for MNO 4 (Figure 8). These required values of spectra for serving data traffic imply that MNO 1 and MNO 2 have a lack of spectrum of 233.3 MHz (i.e., 533.3–300 MHz) and 100 MHz (i.e., 400–300 MHz), respectively. On the other hand, MNO 3 and MNO 4 have an excess spectrum of 33.33 MHz (i.e., 266.67–300 MHz) and 300 MHz (i.e., 0–300 MHz), respectively, as shown in Figure 8.

Hence, by applying the proposed DESA, MNO 1 and MNO 2 can lease the excessive spectra of MNO 3 and MNO 4 using secondary spectrum trading. This can allow both MNO 1 and MNO 2 (who would otherwise suffer from insufficient spectrum) to serve their respective user demands, whereas MNO 3 and MNO 4 (who would otherwise waste their excessive spectrum) can make a profit by leasing their respective spectra to MNO 1 and MNO 2. Hence, the proposed DESA results in an improved spectrum utilization of the 28 GHz spectrum allocated to the country, as well as a win–win situation for each MNO of the country over an agreement time *t*_agg_.

Given the total number of subscribers in the country at the start of the agreement time *t*_agg_, the number of subscribers of each MNO may either increase or decrease after the end of the current agreement time, i.e., *t*_agg_ + 1. Depending on who shares with whom at *t*_agg_, to address the changing user demands of all four MNOs at *t*_agg_ + 1, the leased spectrum to either MNO 1 or MNO 2 or both can be taken back or given away further in an appropriate amount complying with the change in user demand of MNO 3 or MNO 4 or both at *t*_agg_ + 1. In this regard, assume that, at the next agreement term *t*_agg_ + 1, the data traffic demand for MNO 3 and MNO 4 increases, whereas the data traffic demand of MNO 1 and MNO 2 decreases. To address the increased data traffic demands of MNO 3 and MNO 4 at *t*_agg_ + 1, MNO 3 takes its spectrum back from MNO 2 that it leased to MNO 2 at *t*_agg_, whereas MNO 4 leases spectrum from MNO 2, as well as takes a portion of its spectrum back from MNO 1 that it leased to MNO 1 at *t*_agg_, as shown in Figure 8. The redistribution of spectra among MNOs will continue to repeat at each agreement term in the future so long as there exists a mutual understanding among MNOs. Note that, in Figure 8, it can be found that, no matter how the spectrum is redistributed among MNOs at any agreement term, the total spectrum of all MNOs countrywide for serving data traffic remains the same.

Such an on-demand basis update using the proposed DESA among MNOs can be done for a short to medium agreement term *t*_agg_ (e.g., *t*_agg_ could be set to 3–6 months) with a mutual understanding. Hence, rather than considering only a single MNO, adopting all MNOs countrywide for the secondary spectrum trading can help, in terms of, particularly, serve necessary user demands for data traffic by an MNO, gain profit from the unused spectrum of an MNO by leasing its unused spectrum to other MNOs to survive in the competitive market even with a less number of subscribers, and improve the overall 28 GHz mmWave spectrum utilization and the quality of 5G services of all four MNOs in the country.

## 6. Conclusions

In this paper, we propose a dynamic exclusive-use spectrum access (DESA) method to share partly and exclusively the licensed millimeter-wave (mmWave) spectrum of one mobile network operator (MNO) to another of a country in a dynamic and on-demand basis for a certain time of their mutual agreement by exploiting secondary spectrum trading to improve countrywide spectrum utilization. Operating as the light licensing spectrum access method, the proposed DESA method has taken advantage of both the individual authorization and general authorization licensing methods to allow a more flexible, simplified, and interference-protection secured way for sharing the mmWave spectrum of one MNO to another. For a system architecture consisting of an arbitrary number of MNOs in a country each allocated to an equal amount of licensed 28 GHz mmWave spectrum, we formulate the proposed DESA method and deduce the optimal amount of shared spectrum for each MNO, which is updated at each agreement term by presenting an iterative algorithm. We derive average capacity, spectral efficiency, energy efficiency, and cost efficiency performance metrics for all MNOs countrywide and present extensive numerical and simulation results and analyses for an example scenario of a country with four MNOs each assigned statically with an equal amount of 28 GHz mmWave spectrum.

By applying DESA at an agreement term *t*_agg_, it is shown that MNOs with a lack of minimum licensed spectra to serve their data traffic can lease at the cost of payment of the required additional spectra from other MNOs having unused or under-utilized licensed spectra. Moreover, due to sharing the licensed spectrum of one MNO with another, it is presented that the overall countrywide average capacity, spectral efficiency, energy efficiency, and cost efficiency can be improved, respectively, by 25%, 25%, 17.5%, and 20% with applying DESA. Further, we show that, with applying DESA to all MNOs countrywide, the expected spectral efficiency and energy efficiency requirements for 6G mobile systems can be achieved by reusing the same mmWave spectrum to 20% fewer buildings of small cells. In addition, when evaluating MNOs individually, because of leasing the unused spectra, seller MNOs typically require fewer buildings of small cells than that required by buyer MNOs due to operating at their maximum available spectra. Finally, using the statistics of subscribers of all MNOs, we have presented a case study for 5G networks to show the application of the proposed DESA method to an arbitrary country of four MNOs.

## Figures and Tables

**Table 1:** Default parameters and assumptions.

Parameters and Assumptions	Value		
*For all MNOs countrywide*			
Countrywide total of 28 GHz spectrum bandwidth	200 MHz		
Countrywide total number of MNOs and subscribers	4 and $N_{C,\max}$		
Number of subscribers for MNOs 1, 2, 3, and 4, respectively	40%, 30%, 20%, and 10% of $N_{C,\max}$		
Total 28 GHz mmWave spectrum bandwidth and reserved spectrum for each MNO	50 MHz and 10 MHz (for 28 GHz)		
*For each MNO*			
E-UTRA simulation case ^1^	3GPP Case 3		
Cellular layout ^2^, Inter-site distance (ISD) ^1,2^, transmit direction	Hexagonal grid, dense urban, 3 sectors per macrocell site, 1732 m, and downlink		
Carrier frequency ^2,3^	Licensed 2 GHz non-LOS (NLOS) microwave spectrum band for macrocells and picocells, licensed 28 GHz LOS mmWave spectrum band for small cells		
Number of cells	1 macrocell, 2 picocells, 48 small cells per building		
Total BS transmit power ^1^ (dBm)	46 for macrocell ^1,4^, 37 for picocells ^1^, 19 for 28 GHz for small cells ^1,3,4,6^		
Co-channel small-scale fading model ^1,5,6^	Frequency selective Rayleigh for 2 GHz NLOS spectrum for macrocells and picocells, no small-scale fading for 28 GHz LOS spectrum for small cells		
External wall penetration loss ^1^ (*L*_ow_)	20 dB for 2 GHz spectrum		
Path loss	MBS and a UE ^1^	Outdoor macrocell UE	PL(dB) = 15.3 + 37.6log_10_*R*, *R* is in m
		Indoor macrocell UE	PL(dB) = 15.3 + 37.6log_10_*R* + *L*_ow_, *R* is in m
	PBS and a UE ^1^	PL(dB) = 140.7 + 36.7log_10_*R*, *R* is in km	
	SBS and a UE ^1,2,3,5^	PL(dB) = 61.38 + 17.97log_10_*R*, *R* is m	
Lognormal shadowing standard deviation (dB)	8 for MBS ^2^, 10 for PBS ^1^, and 9.9 for 28 GHz LOS spectrum for SBS ^2,3,5^		
Antenna configuration	Single-input single-output for all BSs and UEs		
Antenna pattern (horizontal)	Directional (120°) for MBS ^1^, omnidirectional for PBS ^1^ and SBS ^1^		
Antenna gain plus connector loss (dBi)	14 for MBS ^2^, 5 for PBS ^1^, 5 for SBS ^1,3,6^		
UE antenna gain ^2,3,6^	0 dBi (for 2 GHz), 5 dBi (for 28 GHz, Biconical horn)		
UE noise figure^2,6^ and UE speed ^1^	9 dB (for 2 GHz) and 10 dB (for 28 GHz), 3 km/hr		
Total number of macrocell UEs	30		
Picocell coverage and macrocell UEs offloaded to all picocells ^1^	40 m (radius), 2/15		
Indoor macrocell UEs ^1^	35%		
3D multistory building and SBS models (for regular square-grid structure)	Number of buildings	*L*	
	Number of floors per building	6	
	Number of apartments per floor	8	
	Number of SBSs per apartment	1	
	Total number of SBSs per building	48	
	Area of an apartment	10 × 10 m^2^	
	Location of an SBS in an apartment	Center of the ceiling	
Scheduler and traffic model ^2^	Proportional Fair (PF) and full buffer		
Type of SBSs	Closed Subscriber Group (CSG) femtocell base stations		
Channel State Information (CSI)	Ideal		
TTI^1^, scheduler time constant (*t*_c_), *t*_agg_	1 ms, 100 ms, 6 months		
Total simulation run time	8 ms		

Taken ^1^ from [36], ^2^ from [37], ^3^ from [38], ^4^ from [39], ^5^ from [40], ^6^ from [41].

**Table 2:** Required values of *L* to satisfy both average SE and EE requirements for 6G mobile systems.

MNO	*L* (To Satisfy both Average SE and EE Requirements for 6G Mobile Systems)						
	$\sigma_{\mathrm{SE},o,}^{\mathrm{mmW},t_{\mathrm{agg}}}\left(L\right)\;\geq \sigma_{\mathrm{SE}}^{6G}$		$\sigma_{\mathrm{EE},o}^{\mathrm{mmW},t_{\mathrm{agg}}}\left(L\right)\;\leq \sigma_{\mathrm{EE}}^{6G}$		$\max\left(\sigma_{\mathrm{SE},o}^{\mathrm{mmW},t_{\mathrm{agg}}}\left(L\right),\sigma_{\mathrm{EE},o}^{\mathrm{mmW},t_{\mathrm{agg}}}\left(L\right)\right)$		
	Without DESA	With DESA	Without DESA	With DESA	Without DESA	With DESA	With DESA/Without DESA
1	32	30	1	1	32	30	0.937
2	32	31	1	1	32	31	0.968
3	40	34	1	1	40	34	0.85
4	80	42	1	1	80	42	0.525
All	40	32	1	1	40	32	0.80

## Appendix A

**Table A1:** A list of selected notations.

Notation	Description
$M_{C,\max}$	The total amount of mmWave spectrum in RBs allocated to a country
*O*	Maximum number of MNOs of a country
$t_{\mathrm{agg}}$	An agreement term
*i, t, o,*	Index of an RB, a transmission time interval (TTI), and an MNO, respectively,
*L*	Number of buildings of small cells per macrocell
$N_{o,t_{\mathrm{agg}}}$	Total number of subscribers for an MNO *o* at $t_{\mathrm{agg}}$
$N_{C,\;\max,t_{\mathrm{agg}}}$	Maximum number of subscribers of all MNOs of a country at $t_{\mathrm{agg}}$
$M_{o,t_{\mathrm{agg}}}^{\mathrm{data}}$	Required data traffic spectrum in RBs for an MNO *o* at $t_{\mathrm{agg}}$
$M_{o,t_{\mathrm{agg}}}^{\mathrm{res}}$	Reserved spectrum in RBs of an MNO *o* at $t_{\mathrm{agg}}$
$M_{o,t_{\mathrm{agg}}}^{\mathrm{shared}}$	Shared or leased spectrum in RBs for each MNO *o* at $t_{\mathrm{agg}}$
*M*	An equal amount of licensed mmWave spectrum per MNO in RBs
$M_{\mathrm{MBS},o}$	Operating spectrum of a macrocell for an MNO *o* in RBs
*Q*	Maximum simulation run time in TTI
$\rho_{t,i,o}^{t_{\mathrm{agg}}}$	Received signal-to-interference-plus-noise ratio for a UE at RB = *i* in TTI = *t* for an MNO *o* at $t_{\mathrm{agg}}$
$\sigma_{t,i,o}^{t_{\mathrm{agg}}}\left(\cdot \right)$	A link throughput for a UE at RB = *i* in TTI = *t* for an MNO *o* at $t_{\mathrm{agg}}$
$\sigma_{\mathrm{cap},o}^{\mathrm{sys},t_{\mathrm{agg}}}\left(\cdot \right)$, $\sigma_{\mathrm{SE},o}^{\mathrm{sys},t_{\mathrm{agg}}}\left(\cdot \right)$, $\sigma_{\mathrm{EE},o}^{\mathrm{sys},t_{\mathrm{agg}}}\left(\cdot \right)$	System-level average capacity, spectral efficiency, and energy efficiency, respectively, of an MNO *o* at $t_{\mathrm{agg}}$
$\sigma_{\mathrm{cap},o}^{\mathrm{mmW},t_{\mathrm{agg}}}\left(\cdot \right)$, $\sigma_{\mathrm{SE},o}^{\mathrm{mmW},t_{\mathrm{agg}}}\left(\cdot \right)$, $\sigma_{\mathrm{EE},o}^{\mathrm{mmW},t_{\mathrm{agg}}}\left(\cdot \right)$	Average capacity, spectral efficiency, and energy efficiency, respectively, of the only mmWave enabled small cells of an MNO *o* at $t_{\mathrm{agg}}$
$\sigma_{\mathrm{cap},o,\mathrm{with}\;\mathrm{DESA}}^{\mathrm{mmW},t_{\mathrm{agg}}}\left(\cdot \right)$, $\sigma_{\mathrm{SE},o,\mathrm{with}\;\mathrm{DESA}}^{\mathrm{mmW},t_{\mathrm{agg}}}\left(\cdot \right)$, $\sigma_{\mathrm{EE},o,\mathrm{with}\;\mathrm{DESA}}^{\mathrm{mmW},t_{\mathrm{agg}}}\left(\cdot \right)$	Average capacity, spectral efficiency, and energy efficiency, respectively, for an MNO *o* at $t_{\mathrm{agg}}$ with applying DESA for mmWave enabled small cells only
$\sigma_{\mathrm{cap},o,\mathrm{without}\;\mathrm{DESA}}^{\mathrm{mmW},t_{\mathrm{agg}}}\left(\cdot \right)$, $\sigma_{\mathrm{SE},o,\mathrm{without}\;\mathrm{DESA}}^{\mathrm{mmW},t_{\mathrm{agg}}}\left(\cdot \right)$, $\sigma_{\mathrm{EE},o,\mathrm{without}\;\mathrm{DESA}}^{\mathrm{mmW},t_{\mathrm{agg}}}\left(\cdot \right)$	Average capacity, spectral efficiency, and energy efficiency, respectively, for an MNO *o* at $t_{\mathrm{agg}}$ without applying DESA for mmWave enabled small cells only
$\varsigma_{\mathrm{cap},o,\mathrm{IF}}^{\mathrm{mmW},t_{\mathrm{agg}}}\left(\cdot \right)$, $\varsigma_{\mathrm{SE},o,\mathrm{IF}}^{\mathrm{mmW},t_{\mathrm{agg}}}\left(\cdot \right)$, $\varsigma_{\mathrm{EE},o,\mathrm{IF}}^{\mathrm{mmW},t_{\mathrm{agg}}}\left(\cdot \right)$	Improvement factor in average capacity, spectral efficiency, and energy efficiency, respectively, due to applying DESA for an MNO *o* at $t_{\mathrm{agg}}$
$\varepsilon_{C}$	Cost of the total amount of mmWave spectrum allocated to a country expressed in per Hertz of the licensed spectrum
$\varepsilon_{o}$	Cost of the mmWave spectrum paid by an MNO *o* expressed in per Hertz of the licensed spectrum
$\varsigma_{\mathrm{CE},o,\mathrm{with}\;\mathrm{DESA}}^{\mathrm{mmW},t_{\mathrm{agg}}}\left(\cdot \right)$, $\varsigma_{\mathrm{CE},o,\mathrm{without}\;\mathrm{DESA}}^{\mathrm{mmW},t_{\mathrm{agg}}}\left(\cdot \right)$	Cost efficiency of small cell networks at $t_{\mathrm{agg}}$ for an MNO *o* with applying and without applying DESA, respectively,
$\varsigma_{\mathrm{CE},o,\mathrm{IF}}^{\mathrm{mmW},t_{\mathrm{agg}}}\left(\cdot \right)$	Improvement factor in cost efficiency due to applying DESA for an MNO *o* at $t_{\mathrm{agg}}$
